# Differentially altered social dominance- and cooperative-like behaviors in *Shank2*- and *Shank3*-mutant mice

**DOI:** 10.1186/s13229-020-00392-9

**Published:** 2020-10-30

**Authors:** Kyung Ah Han, Taek Han Yoon, Jungsu Shin, Ji Won Um, Jaewon Ko

**Affiliations:** 1 Department of Brain and Cognitive Sciences, Daegu Gyeongbuk Institute of Science and Technology (DGIST), Daegu, 42988 Korea; 2 Core Protein Resources Center, DGIST, Daegu, 42988 Korea

**Keywords:** Autism, Shank2, Shank3, Social cooperation, Social dominance, Tube test

## Abstract

**Background:**

Recent progress in genomics has contributed to the identification of a large number of autism spectrum disorder (ASD) risk genes, many of which encode synaptic proteins. Our understanding of ASDs has advanced rapidly, partly owing to the development of numerous animal models. Extensive characterizations using a variety of behavioral batteries that analyze social behaviors have shown that a subset of engineered mice that model mutations in genes encoding Shanks, a family of excitatory postsynaptic scaffolding proteins, exhibit autism-like behaviors. Although these behavioral assays have been useful in identifying deficits in simple social behaviors, alterations in complex social behaviors remain largely untested.

**Methods:**

Two syndromic ASD mouse models—*Shank2* constitutive knockout [KO] mice and Shank3 constitutive KO mice—were examined for alterations in social dominance and social cooperative behaviors using tube tests and automated cooperation tests. Upon naïve and salient behavioral experience, expression levels of c-Fos were analyzed as a proxy for neural activity across diverse brain areas, including the medial prefrontal cortex (mPFC) and a number of subcortical structures.

**Findings:**

As previously reported, *Shank2* KO mice showed deficits in sociability, with intact social recognition memory, whereas *Shank3* KO mice displayed no overt phenotypes. Strikingly, the two *Shank* KO mouse models exhibited diametrically opposed alterations in social dominance and cooperative behaviors. After a specific social behavioral experience, Shank mutant mice exhibited distinct changes in number of c-Fos^+^ neurons in the number of cortical and subcortical brain regions.

**Conclusions:**

Our results underscore the heterogeneity of social behavioral alterations in different ASD mouse models and highlight the utility of testing complex social behaviors in validating neurodevelopmental and neuropsychiatric disorder models. In addition, neural activities at distinct brain regions are likely collectively involved in eliciting complex social behaviors, which are differentially altered in ASD mouse models.

## Background

Autism spectrum disorders (ASDs) are a heterogeneous set of neurodevelopmental disorders characterized by deficits in social communication, social interaction, repetitive behaviors and sensory perception [1]. ASDs also exhibit sexual dimorphism, occurring more frequently in males [2, 3]. Importantly, elegant studies using numerous animal models harboring homologous genetic mutations, in conjunction with application of sophisticated behavioral analyses, have contributed enormously to our current understanding of ASD biology [4, 5]. Nevertheless, progress in investigating complex symptoms of ASD patients has lagged, owing to inherent limitation of traditional behavioral analyses. For instance, the lack of emotional reciprocity is frequently observed in ASD patients, who have difficulty in performing a set of executive functions, including impaired theory of mind, imitation, or joint attention of other people [6, 7]. Deficits in these higher cognitive functions, particularly, empathy, are likely to lead to abnormalities in complex social behaviors, such as social competition and cooperation [6, 7]. Indeed, major symptoms of high-functioning autism include empathic incapacity, reactive aggression and impaired complex social behaviors [8, 9]. However, whether these behavioral features are recapitulated in ASD model animals has received limited research attention.

Numerous abnormalities in synaptic genes have been consistently linked to ASDs, underscoring their strong association with ASD pathogenesis [3]. Among other loci, genes encoding members of the Shank (SH3 and multiple ankyrin repeat domains; also known as ProSAP) family of excitatory scaffold proteins, composed of three members (Shank1, Shank2 and Shank3), are among the “hot spots” for ASD pathogenesis [10–12]. Extensive animal studies using various *Shank* knockout (KO) and knock-in (KI) mice have provided convincing support for the concept of “Shankopathies”, which encompass Shank protein deficiency-induced human brain disorders [13]. Intriguingly, various Shank mutant models display pleiotropic physiological, biochemical and behavioral phenotypes that are reminiscent of the phenotypic heterogeneity of ASD patients [11, 12]. In addition, different lines of *Shank2* and *Shank3* mutant mice in which different exons are deleted exhibit a diverse array of cellular phenotypes, suggesting that dysfunction of different Shank splicing isoforms might lead to different ASD-like pathophysiologies [11]. Although a subset of Shank mutant mice was reported to exhibit altered social dominance behaviors, and affected synapse types were partly identified [14], whether these mice also display altered social cooperative behavior remains to be determined.

In the present study, we used Shank2 KO mice (exons 6–7 of *Shank2a* and *Shank2b* targeted; [15]) and Shank3 KO mice (exon 9 of *Shank3a* targeted; [16]) to probe whether these Shank mutant mice exhibit altered social dominance and cooperative behaviors. We found that Shank2 mutant mice exhibited social dominance-like behavior with impaired social interactions, whereas Shank3 mutant mice exhibited socially submissive behavior with normal social interactions. Strikingly, Shank3 mutant mice showed less cooperative behavior, but Shank2 mutant mice exhibited more cooperative behavior. We also found that neurons in a subset of subcortical areas of the Shank mutant mice were differentially activated/inhibited in response to specific salient social stimuli.

## Methods

### Mice

The *Shank2*^*Δ6−7*^ [15] and *Shank3*^*Δ9*^ [16] mutant mice used in the current study were described previously. All mice were on a C57BL/6N background, produced by back-crossing with C57BL/6N WT mice (purchased from Jackson Research Laboratory) for more than six generations. Heterozygous male and female Shank mutant mice were intercrossed to produce homozygous KO and WT littermates. Mice were housed 2–4 animals per cage, and all experiments were conducted in 8–12-week-old male mice. All mouse work was performed at the DGIST Laboratory Animal Resource Center using approved animal protocols.

### Mouse behaviors

All behavioral tests were conducted using WT males or the indicated homozygous *Shank* KO littermate mice obtained from the mating scheme indicated above.

#### Three-chamber test

The three-chamber apparatus is a transparent Plexiglas box with two transparent partitions dividing left, center and right chambers. The three-chamber test itself consisted of three phases. In the first phase, mice were placed in the center chamber of a clean, empty apparatus and allowed to explore the whole apparatus for 10 min. After habituation, an age- and gender-matched stranger mouse (S1) of the same strain that had never been exposed to the test mouse was placed in a wire cage in a side chamber; an empty wire cage, as an inanimate object (O), was placed on the opposite side (for sociability tests). Mice were then allowed to freely explore the apparatus for 10 min. The stranger mouse was randomly introduced into the left or right chamber. In the third phase, another stranger mouse (S2) was introduced into the empty wire cage (for social recognition tests), and the test mouse was allowed to freely explore for 10 min. For each set of experiments, the orientation of the two wire cages containing S1 or S2 (or empty) was counterbalanced. Mouse movements were monitored with a camera equipped with software that provided a real-time readout of the relative locations of mice in the three chambers. The time spent in each chamber and time spent sniffing the wire cage containing S1 or S2 (or empty) were analyzed using EthoVision XT 11.5 software (Noldus, The Netherlands).

#### Social dominance test

The social dominance test was performed using automated tube test equipment manufactured by Noldus. The tube was 60 cm in length with an inner diameter of 3 cm and contained a central gate that divides the tube in half. Mice were trained to pass through the tube by releasing each mouse at alternating ends of the tube and allowing them to run through the tube. This was repeated 6–8 times per day and was continued until mice quickly traversed the tube without hesitation (at least 3 days). After training was completed, dominance test matches began. For these matches, two unfamiliar mice of different genotypes were introduced into either entrance of the tube; when both mice arrived at the center of the tube, the gate was opened, marking the start of the match. The match was terminated when either mouse was pushed out. If both mice still remained in the tube after 2 min, the test was considered a draw. Mouse pair encounters were staged using a round robin design that tested all possible pairs. The same pair of mice was matched three times on the same day, with mouse entry sides counter balanced.

#### Social cooperation test

Social cooperation learning was carried out in a custom-made white Plexiglas box (20 cm wide, 60 cm long, 25 cm high) divided vertically into two lanes by a transparent, perforated divider. Mouse performance was tracked and recorded using an automated video-tracking system equipped with EthoVision XT 11.5 software, providing a real-time readout of the relative location of mice in three predefined zones (Zone A to Zone B to Zone C). During the learning phase, mice were individually habituated to the cooperation maze once for at least 30 min to overcome neophobia. Throughout the cooperative learning period, mice were water-deprived for 6–8 h a day prior to the daily trial to enhance motivation for the task. On the first day of learning and thereafter for 12 consecutive days, each mouse was placed in the maze with the same partner for a 15-min (maximum duration) session. A trial was considered successful when the mice shuttled coordinately between the 3 predefined zones, with a maximum delay of 10 s from the adjacent zone, and arrived together at the end of the maze (Zone C). Upon fulfillment of the computerized algorithm defining the cooperative condition, peristaltic pumps provided a mutual reward (70-μl 20% sucrose drop) through liquid dispensers. Thus, acquiring mutual rewards required coordinated movement from Zone A to Zone C.

### Antibody

A rabbit monoclonal anti-c-Fos antibody (clone 9F6) was obtained from Cell Signaling Technology.

### Immunohistochemistry

After anesthetization, mice were perfused with 4% paraformaldehyde. Brains were then removed, fixed overnight at 4 °C and then cut into coronal sections (40 µm thickness) using a vibratome. Sections were selected according to Paxinos and Franklin’s, The Mouse Brain in Stereotaxic Coordinates (4th edition), and incubated for 2 h at room temperature with 0.5% Triton X-100 containing blocking solution consisting of phosphate-buffered saline (PBS) plus 5% normal donkey serum albumin and 1 mg/ml bovine serum albumin. Consecutive sections were incubated overnight at 4 °C with an anti-c-Fos monoclonal rabbit primary antibody, diluted 1:500 in blocking solution. Sections were subsequently washed twice with PBS (5 min each) and then incubated with Cy3-conjugated secondary antibodies (1:500; Jackson ImmunoResearch, Catalog #711–165-152). Immunoreactive cells across at least three sections were counted bilaterally using a fixed sample window. All images were acquired using an Axio Scan.Z1 slide scanner.

### c-Fos imaging and quantification

After the indicated behavior test, each mouse was returned to its home cage and kept there for 1.5 h to allow maximal c-Fos expression. After preparing coronal brain sections as described above, selected brain sections (≥ 3 slices/mouse) were immunostained using an anti-c-Fos antibody following the indicated immunohistochemistry protocol. Whole selected brain sections were scanned using a slide scanner (Axio Scan.Z1; Carl Zeiss) with a 20 × objective lens; all image settings were kept constant during image acquisition. Z-stack images obtained with the slide scanner were converted to maximal projections, and the acquired images were further processed using ZEN software installed in Axio Scan.Z1. Prior to counting c-Fos-positive immunoreactive puncta, all brain sections were overlaid with the corresponding Allen Mouse Brain Atlas maps using the ImageJ plugin (BigWarp). After clearly distinguishing each anatomical subregion during alignment, the density of c-Fos-positive immunoreactive puncta in each brain region was analyzed in a blinded manner using MetaMorph software (Molecular Devices Corp.). All quantified puncta were within the 9–30-μm diameter range and were thresholded using a preset value.

### Statistics

All data shown are presented as means ± standard error of the mean (SEM). All experiments were performed using at least two independent mouse cohorts and were statistically analyzed with Mann–Whitney *U* test or repeated measures two-way analysis of variance (ANOVA) followed by Sidak’s test, using GraphPad software (San Diego, CA, USA). A *p* value < 0.05 was considered statistically significant. See Additional file 1: Tables S1–S3 for detailed summary.

## Results

Among the more than a dozen previously established Shank mutant mice, two *Shank* KO mice, designated *Shank2*^*Δ6−7*^ (deletion of exons 6 and 7) and *Shank3*^*Δ9*^ (deletion of exon 9), were available to us. *Shank2*^*Δ6−7*^ mice exhibit a number of autistic-like behavioral deficits, whereas *Shank3*^*Δ9*^ mice are outwardly normal with mildly increased repetitive behaviors [15, 16]. Despite these differences, both Shank mouse models display altered excitation–inhibition ratios in neurons of different brain regions [15, 16]. We first confirmed the reported social interaction and social novelty recognition memory phenotypes of these Shank mutant mice by performing three-chamber tests (Additional file 1: Fig. S1). In line with a previous report [16], *Shank2*^*Δ6−7*^ mice exhibited significantly reduced interaction with normal target mice compared with wildtype (WT) control mice, but showed normal social novelty recognition memory (Additional file 1: Fig. S1a–c). In contrast, *Shank3*^*Δ9*^ mice displayed normal levels of social interaction and social novelty recognition (Additional file 1: Fig. S1d–f).

**Fig. 1:**
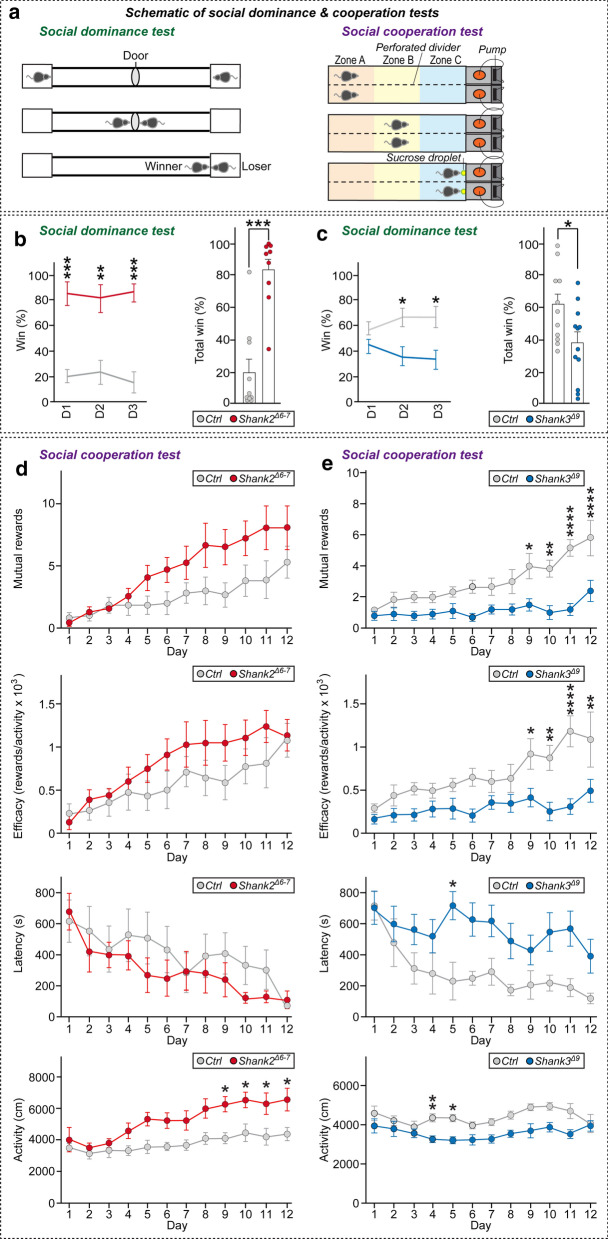
Altered social dominance and social cooperative behaviors of Shank mutant mice. **a** Schematics of tube and social cooperation behavior tests. *Left panel:* Tube test design. *Right panel:* Social cooperation test design. **b**, **c** Analysis of social dominance of *Shank2*^*∆6–7*^ mice (**b**) and *Shank3*^*∆9*^ mice (**c**). Representative graphs showing comparable win percentages for *Shank2*^*∆6–7*^ and *Shank3*^*∆9*^ mice and their littermate controls. Data are expressed as means ± SEMs (*p < 0.05, **p < 0.01, ***p < 0.001; Mann–Whitney *U* test). ‘n’ denotes the number of mice analyzed: Ctrl (**b**), n = 10; *Shank2*^*∆6–7*^ (**b**), n = 9; Ctrl (**c**), n = 11; *Shank3*^*∆9*^ (**c**), n = 13. **d**, **e** Analysis of social cooperation in *Shank2*^*∆6–7*^ mice (**d**) and *Shank3*^*∆9*^ mice (**e**). The performance of *Shank2*^*∆6–7*^ or *Shank3*^*∆9*^ mice and their WT littermates in social cooperation tests was evaluated. Data are expressed as means ± SEMs (n denotes the number of mouse pairs analyzed; n = 6 (Ctrl, **d**); n = 7 (*Shank2*^*∆6–7*^*,* **d**); n = 6 (Ctrl, **e**), n = 10 (*Shank3*^*∆9*^*,* **e**); *p < 0.05, **p < 0.01, ****p < 0.0001; two-way repeated ANOVA). Mutual rewards (Genotype: F_(1,9)_ = 1.583, *p* = 0.2400; Day: F_(3.763, 33.87)_ = 8.463, *p* < 0.0001; Genotype x Day: F_(11, 99)_ = 1.171, *p* = 0.3168, **d**; Genotype: F_(1,14)_ = 25.52, *p* = 0.0002; Day: F_(4.437, 62.12)_ = 7.205, *p* < 0.0001; Genotype x Day: F_(11, 154)_ = 2.641, *p* = 0.0040, **e**), efficacy (Genotype: F_(1,9)_ = 0.4601, *p* = 0.5146; Day: F_(3.994,35.94)_ = 7.386, *p* = 0.0002; Genotype × Day: F_(11,99)_ = 0.6153, *p* = 0.8119, **d**; Genotype: F_(1,14)_ = 23.11, *p* = 0.0003; Day: F_(5.091,71.27)_ = 5.113, *p* = 0.0004; Genotype × Day: F_(11,154)_ = 1.969, *p* = 0.0350, **e**), latency (Genotype: F_(1,9)_ = 0.04543, *p* = 0.8360; Day: F_(4.166,37.49)_ = 5.841, *p* = 0.0008; Genotype × Day: F_(11,99)_ = 0.7166, p = 0.7201, **d**; Genotype: F_(1,14)_ = 16.28, *p* = 0.0012; Day: F_(6.167,86.33)_ = 2.484, *p* = 0.0278; Genotype × Day: F_(11,154)_ = 0.7885, *p* = 0.6512, **e**) and activity (Genotype: F_(1,9)_ = 6.947, *p* = 0.0271; Day: F_(3.226,29.04)_ = 7.820, *p* = 0.0004; Genotype × Day: F_(11,99)_ = 1.418, *p* = 0.1766, **d**; Genotype: F_(11,209)_ = 1.305, *p* = 0.0031; Day: F_(5.485,104.2)_ = 2.674, *p* = 0.0220; and Genotype × Day: F_(11,209)_ = 1.305, *p* = 0.2230, **e**). See also Additional file 1: Table S1 for summary

To determine whether social dominance behavior is altered in Shank mutant mice, we performed a tube test, a well-established behavioral paradigm for assessing social hierarchy in rodents [17] (Fig. 1a, left). For this, 6–8-week-old male Shank mutant mice and WT littermates were group-housed (3–4 mice of the same genotypes per cage) for at least 2 weeks. Before the tube test, all mice were habituated to the tube environment as described in “Methods” section. Mice were considered habituated if, after their training sessions, they traversed the tube without hesitation (Fig. 1a, left). After habituation and training, WT mice and mice of each mutant Shank model were matched three times over 3 days (designated D1, D2 and D3), as outlined in Figure S2. To avoid the circumstance in which mice are exhausted by repeated matches, we ensured that the same mice are not matched consecutively by designing mouse matches so as to randomize the test order (Additional file 1: Fig. S2). During the training session, mice of both Shank models showed a comparable ability to enter the tube, reach the middle door and reach the opposite entrance side (Additional file 1: Fig. S2). Surprisingly, tube tests between WT versus *Shank2*^*Δ6−7*^ mice and WT versus *Shank3*^*Δ9*^ mice revealed opposite social dominance behaviors in the two genetic models (Fig. 1b, c). *Shank2*^*Δ6−7*^ mice showed a much higher probability of winning matches when tested against WT littermate opponents, whereas *Shank3*^*Δ9*^ mice displayed significantly less dominant behavior in the tube test (Fig. 1b, c). Notably, the dominant or subordinate behaviors of Shank mutant mice became intensified over successive testing days (Fig. 1b, c). Moreover, *Shank2*^*Δ6−7*^ mice took less time to enter the tube and reach the middle door than their WT littermate opponents, partly owing to their hyperactivity, whereas the behavioral performances of *Shank3*^*Δ9*^ mice in entering the tube and reaching the middle door was comparable to that of WT opponents (Additional file 1: Fig. S2).

To determine whether the Shank mutant mice employed in the present study also exhibited other types of complex social behavior, we set up a behavioral paradigm designed to measure social cooperative behavior that was recently applied to WT rats [18]. For this, we built a white, lusterless Plexiglass box divided vertically into two lanes by a transparent, perforated divider and installed a video camera linked to EthoVision software (Fig. 1a, right). If a pair of mice exhibits cooperative movement from the distal area of the maze (termed Zone A) to the reward zone (termed Zone C) in accordance with the installed computer algorithm, a mutual reward (20% sucrose droplet) was automatically provided through liquid dispensers (Fig. 1a, right). Each pair of WT mice or pair of Shank mutant mice was habituated for at least 30 min per day before the actual behavioral analyses and was water-deprived for 6–8 h prior to tests. The next day, each pair of mice was placed in Zone A of the cooperation maze and the number of mutual rewards as well as activity (defined as total distance moved during the recording session), efficacy (defined as number of rewards divided by activity) and latency (defined as time to receive the first reward) were measured during a video-recorded 15-min trial. This analysis was repeated on 12 consecutive days. Similar to results obtained in rats, pairs of WT mice showed a significant increase in the number of mutual rewards and efficacy over successive days during the learning period (Fig. 1d, e). In addition, there was a gradual increase in activity until day 8 that was maintained until day 12, but with a decrease in the latency of daily initial transition from Zone A to C (Fig. 1d, e). *Shank2*^*Δ6−7*^ mice exhibited a trend (though not significant) towards improved performance compared with their WT littermates, in association with higher activity and elevated efficacy (Fig. 1d), suggesting similar levels of motivated behavior between WT and *Shank2*^*Δ6−7*^ mice. In contrast, *Shank3*^*Δ9*^ mice exhibited decreased social cooperative behavior, as reflected in a decrease in the number of mutual rewards and efficacy and reduced activity, although latency was unchanged (Fig. 1e). Overall, these results suggest that these two different Shank mutant mouse models display distinctly altered social dominance and social cooperation behaviors that are not correlated with abnormalities in sociability or social novelty recognition.

**Fig. 2:**
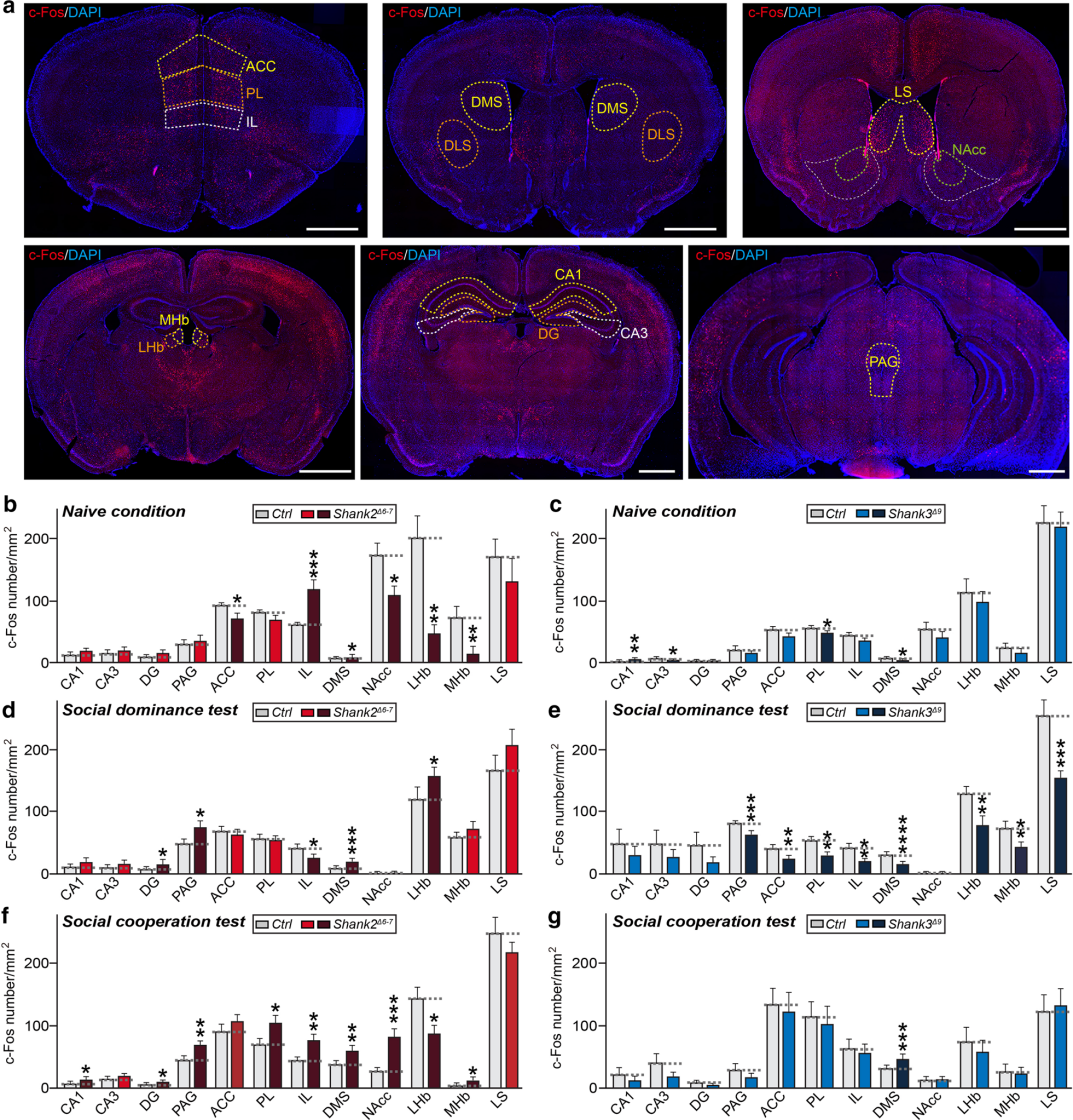
Identification of brain regions exhibiting distinct changes in neuronal activity upon social experience stimulation. **a** Representative images of c-Fos immunostaining in coronal brain sections from the indicated brain regions of control (Ctrl) or the indicated Shank mutant mice. Brain sections were obtained from mice housed in the naïve state or 1.5 h after tube tests or 1.5 h after social cooperation tests. Scale bar: 1 mm. **b–g** Quantification of activated neurons in the mPFC (ACC, PL and IL), striatum (DMS), nucleus accumbens (NAcc), habenula (MHb and LHb), lateral septum (LS), hippocampus (CA1, CA3 and DG) and periaqueductal gray (PAG) by c-Fos immunostaining in *Shank2*^*∆6–7*^ (**b**, **d**, **f**) and *Shank3*^*∆9*^ (**c**, **e**, **g**) mice. Both types of Shank mutant mice were compared with their respective WT littermates (Ctrl). (Ctrl/naïve, n = 11–24 slices (3 mice), *Shank2*^*∆6–7*^/naïve, n = 10–24 slices (3 mice); Ctrl/SD, n = 10–24 slices (4 mice), *Shank2*^*∆6–7*^/SD, n = 10–24 slices (4 mice); Control/SC, n = 11–24 slices (4 mice), *Shank2*^*∆6–7*^/SC, n = 9–24 slices (4 mice); Control/naïve, n = 9–13 slices (3 mice), *Shank3*^*∆9*^/naïve, n = 9–16 slices (4 mice); Control/SD, n = 9–24 slices (4 mice), *Shank3*^*∆9*^/SD, n = 11– 24 slices (4 mice); Control/SC, n = 12–24 slices (4 mice); and *Shank3*^*∆9*^/SC, n = 12–24 slices (4 mice); *p < 0.05, **p < 0.01, ***p < 0.001, ****p < 0.0001; Mann–Whitney *U* test). Ctrl versus *Shank2*^*∆6–7*^/naïve/CA1, *p* = 0.0845; Ctrl versus *Shank2*^*∆6–7*^/naïve/CA3, *p* = 0.6047; Ctrl versus *Shank2*^*∆6–7*^/naïve/DG, *p* = 0.2230; Ctrl versus *Shank2*^*∆6–7*^/naïve/PAG, *p* = 0.9229; Ctrl versus *Shank2*^*∆6–7*^/naïve/ACC, *p* = 0.0222; Ctrl versus *Shank2*^*∆6–7*^/naïve/PL, *p* = 0.1987; Ctrl versus *Shank2*^*∆6–7*^/naïve/IL, *p* = 0.0008; Ctrl versus *Shank2*^*∆6–7*^/naïve/DMS, *p* = 0.0355; Ctrl vs *Shank2*^*∆6–7*^/naïve/NAcc, *p* = 0.0218; Ctrl vs *Shank2*^*∆6–7*^/naïve/LHb, *p* = 0.0020; Ctrl versus *Shank2*^*∆6–7*^/naïve/MHb, *p* = 0.0053; Ctrl versus *Shank2*^*∆6–7*^/naïve/LS, *p* = 0.0914; Ctrl versus *Shank2*^*∆6–7*^/SD/CA1, *p* = 0.2189; Ctrl versus *Shank2*^*∆6–7*^/SD/CA3, *p* = 0.6815; Ctrl versus *Shank2*^*∆6–7*^/SD/DG, *p* = 0.0403; Ctrl versus *Shank2*^*∆6–7*^/SD/PAG, *p* = 0.0295; Ctrl versus *Shank2*^*∆6–7*^/SD/ACC, *p* = 0.6505; Ctrl versus *Shank2*^*∆6–7*^/SD/PL, *p* = 0.7399; Ctrl versus *Shank2*^*∆6–7*^/SD/IL, *p* = 0.0129; Ctrl versus *Shank2*^*∆6–7*^/SD/DMS, *p* = 0.0005; Ctrl versus *Shank2*^*∆6–7*^/SD/NAcc, *p* = 0.4492; Ctrl versus *Shank2*^*∆6–7*^/SD/LHb, *p* = 0.0464; Ctrl versus *Shank2*^*∆6–7*^/SD/MHb, *p* = 0.4567; Ctrl versus *Shank2*^*∆6–7*^/SD/LS, *p* = 0.2657; Ctrl versus *Shank2*^*∆6–7*^/SC/CA1, *p* = 0.0278; Ctrl versus *Shank2*^*∆6–7*^/SC/CA3, *p* = 0.0992; Ctrl versus *Shank2*^*∆6–7*^/SC/DG, *p* = 0.0143; Ctrl versus *Shank2*^*∆6–7*^/SC/PAG, *p* = 0.0013; Ctrl versus *Shank2*^*∆6–7*^/SC/ACC, *p* = 0.0977; Ctrl versus *Shank2*^*∆6–7*^/SC/PL, *p* = 0.0134; Ctrl versus *Shank2*^*∆6–7*^/SC/IL, *p* = 0.0019; Ctrl versus *Shank2*^*∆6–7*^/SC/DMS, *p* = 0.0058; Ctrl versus *Shank2*^*∆9*^/SC/NAcc, *p* = 0.0003; Ctrl versus *Shank2*^*∆6–7*^/SC/LHb, *p* = 0.0098; Ctrl versus *Shank2*^*∆6–7*^/SC/MHb, *p* = 0.0482; Ctrl versus *Shank2*^*∆6–7*^/SC/LS, *p* = 0.4863; Ctrl versus *Shank3*^*∆9*^/naïve/CA1, *p* = 0.0093; Ctrl versus *Shank3*^*∆9*^/naïve/CA3, *p* = 0.0225; Ctrl versus *Shank3*^*∆9*^/naïve/DG, *p* = 0.5499; Ctrl versus *Shank3*^*∆9*^/naïve/PAG, *p* = 0.6771; Ctrl versus *Shank3*^*∆9*^/naïve/ACC, *p* = 0.0877; Ctrl versus *Shank3*^*∆9*^/naïve/PL, *p* = 0.0214; Ctrl versus *Shank3*^*∆9*^/naïve/IL, *p* = 0.0915 ; Ctrl versus *Shank3*^*∆9*^/naïve/DMS, *p* = 0.0225; Ctrl versus *Shank3*^*∆9*^/naïve/NAcc, *p* = 0.2523; Ctrl versus *Shank3*^*∆9*^/naïve/LHb, *p* = 0.7103; Ctrl versus *Shank3*^*∆9*^/naïve/MHb, *p* = 0.3693; Ctrl versus *Shank3*^*∆9*^/naïve/LS, *p* = 0.9279; Ctrl versus *Shank3*^*∆9*^/SD/CA1, *p* = 0.1978; Ctrl versus *Shank3*^*∆9*^/SD/CA3, *p* = 0.1600; Ctrl versus *Shank3*^*∆9*^/SD/DG, *p* = 0.2189; Ctrl versus *Shank3*^*∆9*^/SD/PAG, *p* = 0.0006; Ctrl versus *Shank3*^*∆9*^/SD/ACC, *p* = 0.0018; Ctrl versus *Shank3*^*∆9*^/SD/PL, *p* = 0.0001; Ctrl versus *Shank3*^*∆9*^/SD/IL, *p* = 0.0014; Ctrl versus *Shank3*^*∆9*^/SD/DMS, *p* < 0.0001; Ctrl versus *Shank3*^*∆9*^/SD/NAcc, *p* = 0.4746; Ctrl versus *Shank3*^*∆9*^/SD/LHb, *p* = 0.0099; Ctrl versus *Shank3*^*∆9*^/SD/MHb, *p* = 0.0036; Ctrl versus *Shank3*^*∆9*^/SD/LS, *p* = 0.0001; Ctrl versus *Shank3*^*∆9*^/SC/CA1, *p* = 0.8428; Ctrl versus *Shank3*^*∆9*^/*SC*/CA3, *p* = 0.1600; Ctrl versus *Shank3*^*∆9*^/SC/DG, *p* = 0.7125; Ctrl versus *Shank3*^*∆9*^/SC/PAG, *p* = 0.1600; Ctrl versus *Shank3*^*∆9*^/SC/ACC, *p* = 0.1432; Ctrl versus *Shank3*^*∆9*^/SC/PL, *p* = 0.1135; Ctrl versus *Shank3*^*∆9*^/SC/IL, *p* = 0.5137; Ctrl versus *Shank3*^*∆9*^/SC/DMS, *p* = 0.0004; Ctrl versus *Shank3*^*∆9*^/SC/NAcc, *p* = 0.0841; Ctrl versus *Shank3*^*∆9*^/SC/LHb, *p* = 0.5137; Ctrl versus *Shank3*^*∆9*^/SC/MHb, *p* = 0.7985; and Ctrl versus *Shank3*^*∆9*^/SC/LS, *p* = 0.6707. See also Additional file 1: Table S2 for summary

Lastly, to understand how different types of social experiences influence neuronal activities, we examined the levels of c-Fos, a marker of neuronal activation, in 12 selected brain regions that are possibly linked to social dominance and/or social cooperative behavior in mice [19] (Fig. 2). Both WT and Shank mutant mice were subjected to either tube tests or social cooperation tests and then sacrificed after the last round of experiments and subjected to quantitative immunohistochemical analyses (Fig. 2). A parallel cohort of both WT and Shank mutant mice not subjected to any behavioral tests (i.e., “naïve”) was also included (Fig. 2b, c and Additional file 1: Figs. S3 and S4). Under naïve conditions, c-Fos-positive puncta densities in *Shank2*^*Δ6−7*^ brains were significantly decreased compared with those in WT mice in a variety of brain regions, including the anterior cingulate cortex (ACC), nucleus accumbens core (NAcc), lateral habenula (LHb) and medial habenula (MHb) (Fig. 2b and Additional file 1: Fig. S3). Intriguingly, the infralimbic cortex (IL) and dorso-medial striatum (DMS) showed a marked increase in c-Fos-positive puncta density in *Shank2*^*Δ6−7*^ mice under naïve conditions (Fig. 2b and Additional file 1: Fig. S3). In contrast, discrete brain regions of *Shank3*^*Δ9*^ mice displayed distinctively altered c-Fos levels under naïve conditions, namely upregulation in the hippocampal CA1 region, but downregulation in the hippocampal CA3 region, prelimbic cortex (PL) and DMS (Fig. 2c and Additional file 1: Fig. S4). Remarkably, *Shank2*^*Δ6−7*^ and *Shank3*^*Δ9*^ mice exhibited completely different patterns of changes in neuronal activity upon social dominance testing (Fig. 2d, e and Additional file 1: Figs. S3 and S4). In some brain areas, such as the periaqueductal gray (PAG), DMS and LHb, *Shank2*^*Δ6−7*^ and *Shank3*^*Δ9*^ mice exhibited opposite changes in c-Fos-positive puncta densities, implying that the opposite behavioral phenotypes of the two Shank mutant mouse models might be associated with altered neural circuit activities involving these brain regions. Notably, c-Fos-positive puncta intensities across many brain regions were downregulated in *Shank2*^*Δ6−7*^ (but not *Shank3*^*Δ9*^) mice under naïve conditions, likely owing to the extremely hyperactive behaviors of the *Shank2*^*Δ6−7*^ mice (Additional file 1: Fig. S5). Upon social cooperation testing, *Shank2*^*Δ6−7*^ mice displayed a slightly different pattern of neuronal activation in the examined brain regions (Fig. 2f and Additional file 1: Fig. S3). For instance, neuronal activities of PL, IL, PAG, DMS, Nacc and medial habenula (MHb) neurons in *Shank2*^*Δ6−7*^ brains were increased compared with those in WT brains, whereas the neuronal activity of LHb neurons in *Shank2*^*Δ6−7*^ brains was decreased compared with that in WT brains (Fig. 2f). In contrast, neuronal activity upon social cooperation testing was generally similar in *Shank3*^*Δ9*^ mice and WT mice (Fig. 2g). These data suggest that neuronal activities in the mPFC and a subset of subcortical regions underlie different complex social behaviors, necessitating a further dissection of the network of neurons across diverse brain regions involved in eliciting social dominance and/or cooperative behaviors.

## Discussion

In the present study, we applied two behavioral paradigms to measure social dominance and social cooperation ability in two previously reported ASD mutant mice in which the *Shank2* or *Shank3* gene was deleted. Extensive human genetic studies have consistently linked various rare genetic susceptibility variants of both *Shank2* and *Shank3* to ASDs [11, 12]. Moreover, diverse pathophysiological mechanisms underlying Shankopathies, such as abnormalities in NMDA-type glutamate receptor functions, have been identified at synaptic and neural circuit levels in a number of animal models, including mice, rats and macaques [12]. By comparison, behavioral analyses, particularly those examining the face validity of a plethora of ASD mutant animals, have failed to keep pace. In validating new ASD models or deciphering underlying molecular/cellular mechanisms, most, if not all, studies have analyzed conventional social behaviors (e.g., reciprocal social interaction and/or social novelty recognition) using primarily standard behavioral assays in conjunction with systems neuroscience approaches. Puzzlingly, targeting one synaptic gene (e.g., *Shank2* or *Shank3*) using different genetic strategies results in strikingly different and heterogeneous behavioral phenotypes [11, 12]. These results illustrate that differential gene dosages and/or extensive splicing events can act in a complicated manner to contribute to widely varying behavioral phenotypes of Shank mutant mice.

Only a few prior studies have investigated complex social behavior tasks in ASD-associated mutant mice, with one reporting alterations of such behaviors in these mice [14]. In the current study, we found that different ASD mouse models, namely *Shank2*^*Δ6−7*^ and *Shank3*^*Δ9*^, displayed opposite behaviors in social dominance and cooperation tests (Fig. 1). Importantly, both types of Shank mutant mice were normal with respect to social recognition (Additional file 1: Fig. S1), suggesting that impaired perceptual modalities are not involved in abnormalities in social dominance and cooperative behaviors. A majority of male *Shank2*^*Δ6−7*^ mice exhibited frequent aggressive behaviors and heightened anxiety level, as previously reported [15], likely accounting for the increased dominance in the tube test (Fig. 1). However, whether hyperactive and aggressive behavioral traits are linked cooperative behavior of *Shank2*^*Δ6−7*^ mice is uncertain, especially given that the increase in cooperative behavior observed in these mice did not reach statistical significance (Fig. 1). Moreover, *Shank2*^*Δ6−7*^ mice were previously reported to exhibit reduced social communication in the form of ultrasonic vocalizations [15, 20], a finding that does not appear to align with their dominant behaviors in the tube test [21]. Furthermore, despite their overall normal behavioral performances [16], *Shank3*^*Δ9*^ mice displayed less dominant and cooperative behaviors in the current study. At this point, we are unable to explain why different Shank mutant mice exhibit completely opposite behaviors in complex social behavioral tasks. More detailed analyses to pinpoint related behavioral traits, such as fear learning and memory, may provide additional clues [22]. We suppose that an imbalance in excitation/inhibition ratios in a subset of brain regions, including mPFC subnuclei, collectively yield different behavioral phenotypes in different Shank mutant mice. Some previous reports have provided evidence that appropriate excitatory synaptic transmission in mPFC neurons is crucial for normal social dominance behavior [14, 19, 21]. However, both *Shank2*^*Δ6−7*^ and *Shank3*^*Δ9*^ adult mice exhibit decreased excitatory synaptic transmission in mPFC neurons [16, 23]. Consistent with this, alterations in neuronal activity in mPFC subnuclei were not clearly correlated with social dominance behavioral patterns (Fig. 2), suggesting that other mechanisms must be involved in maintaining normal social hierarchy in mice. In the case of social cooperative behaviors, no definitive cellular or molecular mechanisms are yet known. Intriguingly, different social behavioral experiences led to different patterns of neuronal activation (or suppression) in *Shank3*^*Δ9*^ mice (Fig. 2e, g). Only 12 brain regions were chosen for analyses in the current study; thus, a brain-wide investigation of subcortical structures using various ASD mouse models would help to relate specific cell-type activities with complex social behavioral outcomes, possibly in context of various environmental conditions, such as chronic social isolation stress [24]. In sum, our study provides a useful behavioral platform for testing cooperative behavioral alterations in various mouse models of neurodevelopmental disorders, including ASDs.

## Limitations

Our study has several limitations. First, generalizing the central conclusions of the current study will require testing more types of ASD mutant mice, and by extension, a variety of neurodevelopmental disease mouse models, for altered social dominance and/or social cooperation behaviors, as described in the current study. It is expected that other Shank mutant mice might exhibit differentially altered complex social behaviors, as already exemplified for other behavioral paradigms [11, 12]. Thus, large-scale analyses will provide insights into how different Shank mutations contribute to different behavioral phenotypes, which, in turn, should be mechanistically explained using sophisticated molecular and cellular studies. Second, it is unclear how connections of mPFC neurons with neurons in other brain areas collectively shape social dominance and social cooperation behaviors. Optogenetic/chemogenetic approaches designed to precisely dissect specific cell types in the affected brain regions upon designated complex social behaviors should be helpful in this regard. Third, the current study employed only male Shank mutant mice in analyses. Given the well-known sexual dimorphism among individuals with ASD and a subset of ASD mouse models, it would be worthwhile investigating whether alterations in social dominance/cooperation observed in male Shank mutant mice are similarly recapitulated in their female counterparts. Although female rats exhibit more cooperative behavior [18], whether female mice similarly perform better in cooperative behavior tests needs to be validated. Lastly, the relevance of social dominance and/or cooperation to other ASD-relevant social abnormalities should be elucidated.

## Conclusions

The major observations of the current study are that social dominance and social cooperation behaviors are differentially altered in two different Shank mutant mice that were previously established as syndromic ASD mouse models. Our results also demonstrate that neuronal activities in the distinct sets of subcortical structures are differentially responsive to different behavioral experiences. It is likely that imbalanced excitation/inhibition ratios in a subset of brain regions collectively contribute to the abnormalities in social dominance and social cooperation observed in Shank mutant mice. On the basis of our results, we propose that tube tests and social cooperation tests be used as standard assays to validate ASD mouse models. Further studies are clearly required to more fully understand how distinct patterns of mPFC neuronal activity orchestrate representations of diverse social behaviors and how abnormalities of these behaviors are manifested in ASD animal models.

### Supplementary information

**Additional file 1: Figure S1**. Analysis of Shank2∆6-7 and Shank3∆9 mice in the three-chamber test.** Figure S2**. Matching strategy and analyzed parameters in social dominance tests.** Figure S3**. Representative images showing c-Fos immunostaining in the indicated brain regions of Shank2∆6-7 mice.** Figure S4**. Representative images of c-Fos immunostaining in the indicated brain regions of Shank3∆9 mice.** Figure S5**. Quantitative analyses of c-Fos–positive puncta intensity across 12 brain regions of Shank2∆6 7 and Shank3∆9 mice.** Table S1**. Details on statistics for behavioral analyses presented in Figure 1 and S1.** Table S2**. Details on statistics for c-Fos puncta density analyses.** Table S3**. Details on statistics for c-Fos puncta intensity analyses.

## Data Availability

All data generated or analyzed during this study are included in this published article and additional files. Any additional information related to the current study is available from the corresponding author on reasonable request.

## Abbreviations

ACC: Anterior cingulate cortex
ASD: Autism spectrum disorders
CA1: Cornu ammonis 1
CA3: Cornu ammonis 3
CNV: Copy number variation
DG: Dentate gyrus
DMS: Dorso-medial striatum
Hb: Habenula
IL: Infralimbic cortex
LHb: Lateral habenula
LS: Lateral septum
MHb: Medial habenula
mPFC: Medial prefrontal cortex
NAcc: Nucleus accumbens core
PAG: Periaqueductal gray
PL: Prelimbic cortex
Shank2: SH3 and multiple ankyrin repeat domains 2
Shank3: SH3 and multiple ankyrin repeat domains 3
WT: Wild type
