# Correction: Differentially altered social dominance- and cooperative-like behaviors in *Shank2*- and *Shank3*-mutant mice

**DOI:** 10.1186/s13229-024-00612-6

**Published:** 2024-07-30

**Authors:** Kyung Ah Han, Taek Han Yoon, Jungsu Shin, Ji Won Um, Jaewon Ko

**Affiliations:** 1 Department of Brain and Cognitive Sciences, Daegu Gyeongbuk Institute of Science and Technology (DGIST), Daegu, 42988 Korea; 2 Core Protein Resources Center, DGIST, Daegu, 42988 Korea

**Correction to**: *Molecular Autism*** (2020) 11:87.**

10.1186/s13229-020-00392-9.

In this article, the authors note that errors were inadvertently introduced in Additional file 1, owing to a lapse in attention during final figure preparation prior to submission. Duplicated images were erroneously presented in Figure S3 (an LS section from control mice under naive condition and an LS section from *Shank2*^∆6–7^-KO mice under social cooperation test condition). In addition, thorough reanalysis of all cropped images of the whole-brain sections from Control, *Shank2*^∆6–7^-KO, and *Shank3*^∆9^-KO mice revealed that the representative images in Figure S3 (NaCC section from *Shank2*^∆6–7^-KO mice under social dominance test conditions) and Figure S4 (NaCC section from Control and *Shank3*^∆9^-KO mice under social dominance test conditions) were incorrect. These errors partly stemmed from incorrect cropping of the intended brain regions from a single whole-brain section. Note that, in some cases, the presentation of overlapping representative images denoted as different brain subregions is intentional. This is because these images were of the same whole-brain section and the subregions were anatomically adjacent to each other. These corrections do not alter the results or conclusions reported in the paper.

Additional file 1 has been replaced by the corrected version in the original article, and the corrected version of Addition file 1 is attached to this Correction.

### Electronic supplementary material

Below is the link to the electronic supplementary material.

**Additional file 1: Figure S1**. Analysis of *Shank2*^Δ6–7^ and *Shank*^3Δ9^ mice in the three-chamber test. **Figure S2.** Matching strategy and analyzed parameters in social dominance tests. **Figure S3.** Representative images showing c-Fos immunostaining in the indicated brain regions of Shank2^Δ6–7^ mice. **Figure S4.** Representative images of c-Fos immunostaining in the indicated brain regions of Shank3^Δ9^ mice. **Figure S5.** Quantitative analyses of c-Fos–positive puncta intensity across 12 brain regions of Shank2^Δ6–7^ and Shank3^Δ9^ mice. **Table S1**. Details on statistics for behavioral analyses presented in Fig. 1 and S1. **Table S2.** Details on statistics for c-Fos puncta density analyses. **Table S3.** Details on statistics for c-Fos puncta intensity analyses.

