# Supplementary material for: Differentially altered social dominance- and cooperative-like behaviors in Shank2- and Shank3-mutant mice
Source: Mol Autism. 2020 Oct 30;11:87. doi: 10.1186/s13229-020-00392-9 (PMC7602353; doi:10.1186/s13229-020-00392-9)
Supplement: Supplementary file 1 — Additional file 1: Figure S1. Analysis of Shank2∆6-7 and Shank3∆9 mice in the three-chamber test. Figure S2. Matching strategy and analyzed parameters in social dominance tests. Figure S3. Representative images showing c-Fos immunostaining in the indicated brain regions of Shank2∆6-7 mice. Figure S4. Representative images of c-Fos immunostaining in the indicated brain regions of Shank3∆9 mice. Figure S5. Quantitative analyses of c-Fos–positive puncta intensity across 12 brain regions of Shank2∆6 7 and Shank3∆9 mice. Table S1. Details on statistics for behavioral analyses presented in Figure 1 and S1. Table S2. Details on statistics for c-Fos puncta density analyses. Table S3. Details on statistics for c-Fos puncta intensity analyses. [file 13229_2020_392_MOESM1_ESM.doc]

**Additional file 1**


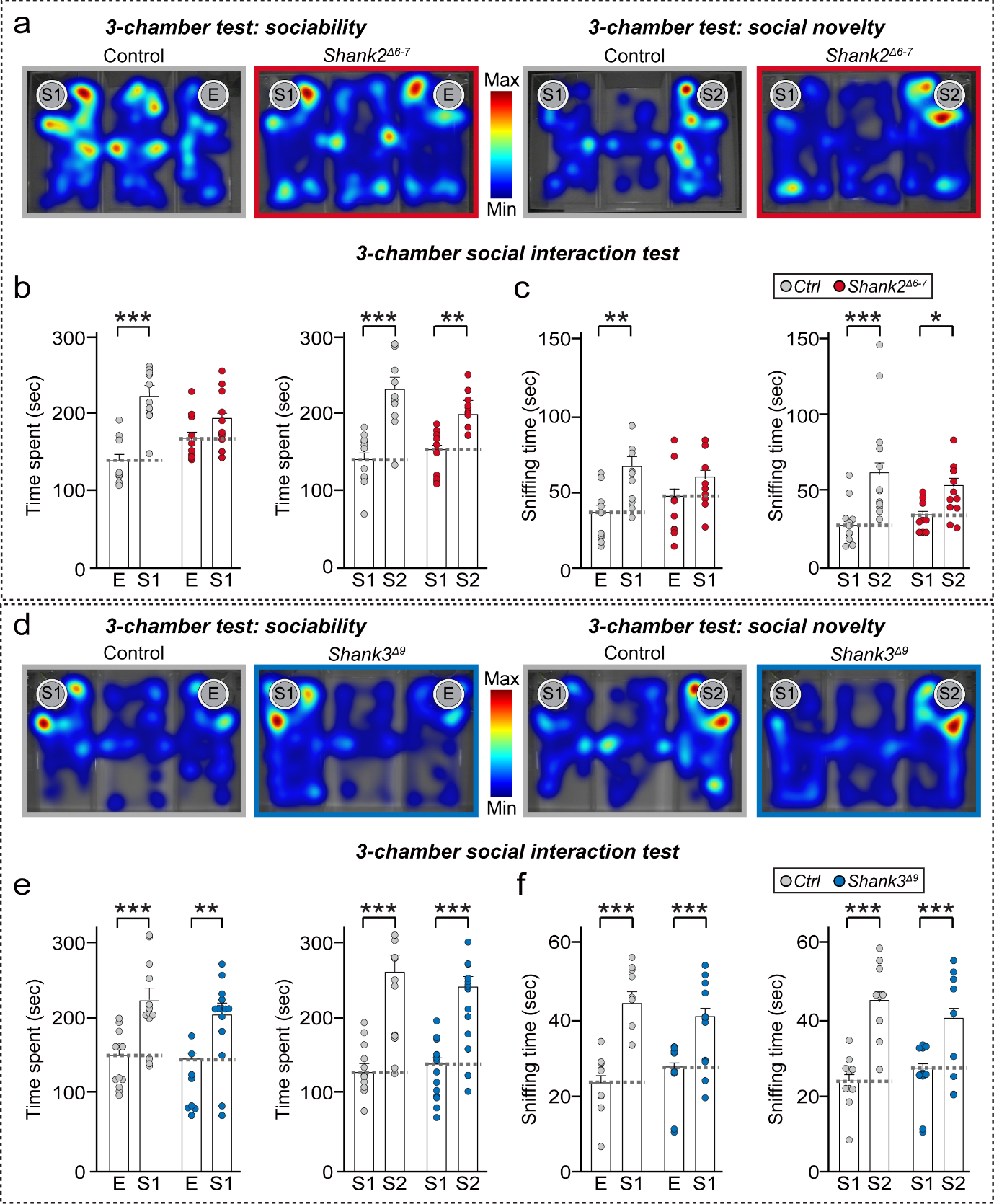


**Figure S1.** Analysis of *Shank2^∆6-7^* and *Shank3^∆9^* mice in the three-chamber test. **a, d,** Representative heat map images for *Shank2*^∆6-7^ (**a**) and *Shank3*^∆9^ (**d**). **b, c, e, f,** Quantification of sociability (**b, e**) and social novelty recognition memory (**c, f**). Sniffing time was defined as the time spent sniffing the novel mouse or the novel object. Data are expressed as means ± SEMs (**p* < 0.05, ***p* < 0.01, ****p* < 0.0001; Mann Whitney U-test). ‘n’ denotes the number of mice analyzed: Ctrl (**b**, **c**), n = 11; *Shank2*^∆6-7^ (**b**, **c**), n = 11; Ctrl (**e**, **f**), n = 19; *Shank3*^∆9^ (**e**, **f**), n = 24. Abbreviations: E, empty cup; S1, stranger mouse 1; S2, stranger mouse 2.


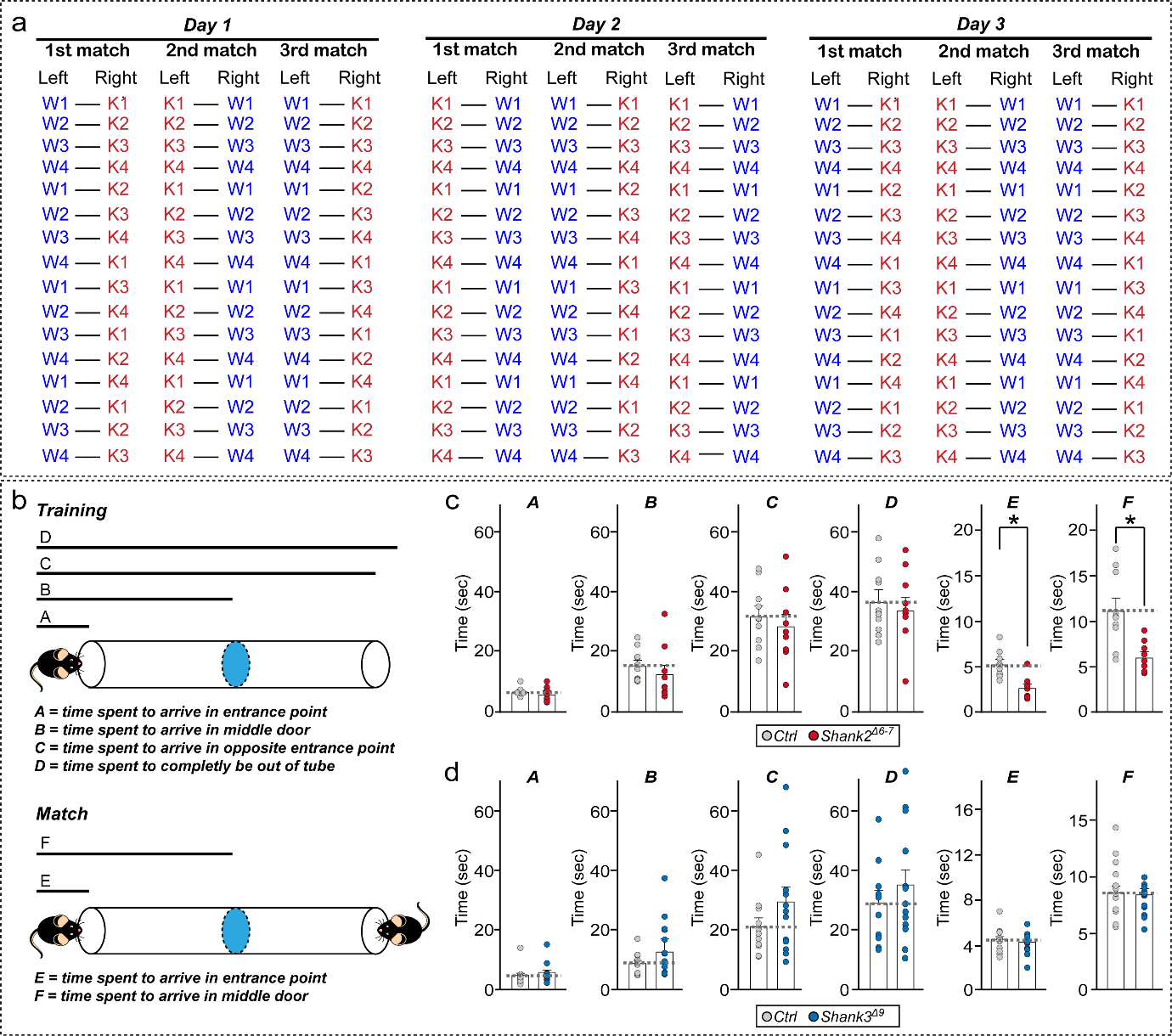


**Figure S2.** Round robin matching strategy and analyzed parameters in social dominance tests. **a,** Schematic diagram of round robin matching strategy for tube tests. Tests were performed three times a day for 3 consecutive days. Matches were performed so as to avoid matching the same mouse in succession. **b,** Schematics of analyzed parameters of tube tests during training and match period. **c, d,** Quantification of time spent by *Shank2^∆6-7^* (**c**) and *Shank3^∆9^* (**d**) mice in reaching the entrance point, middle door and opposite entrance point, and completely exiting the tube, during training and match periods. Data are expressed as means ± SEMs (**p* < 0.05; Mann-Whitney *U* test). ‘n’ denotes the number of mice analyzed: Ctrl (**c**), n = 10; *Shank2^∆6-7^* (**c**), n = 9; Ctrl, (**d**), n = 11; *Shank3^∆9^* (**d**), n = 13.


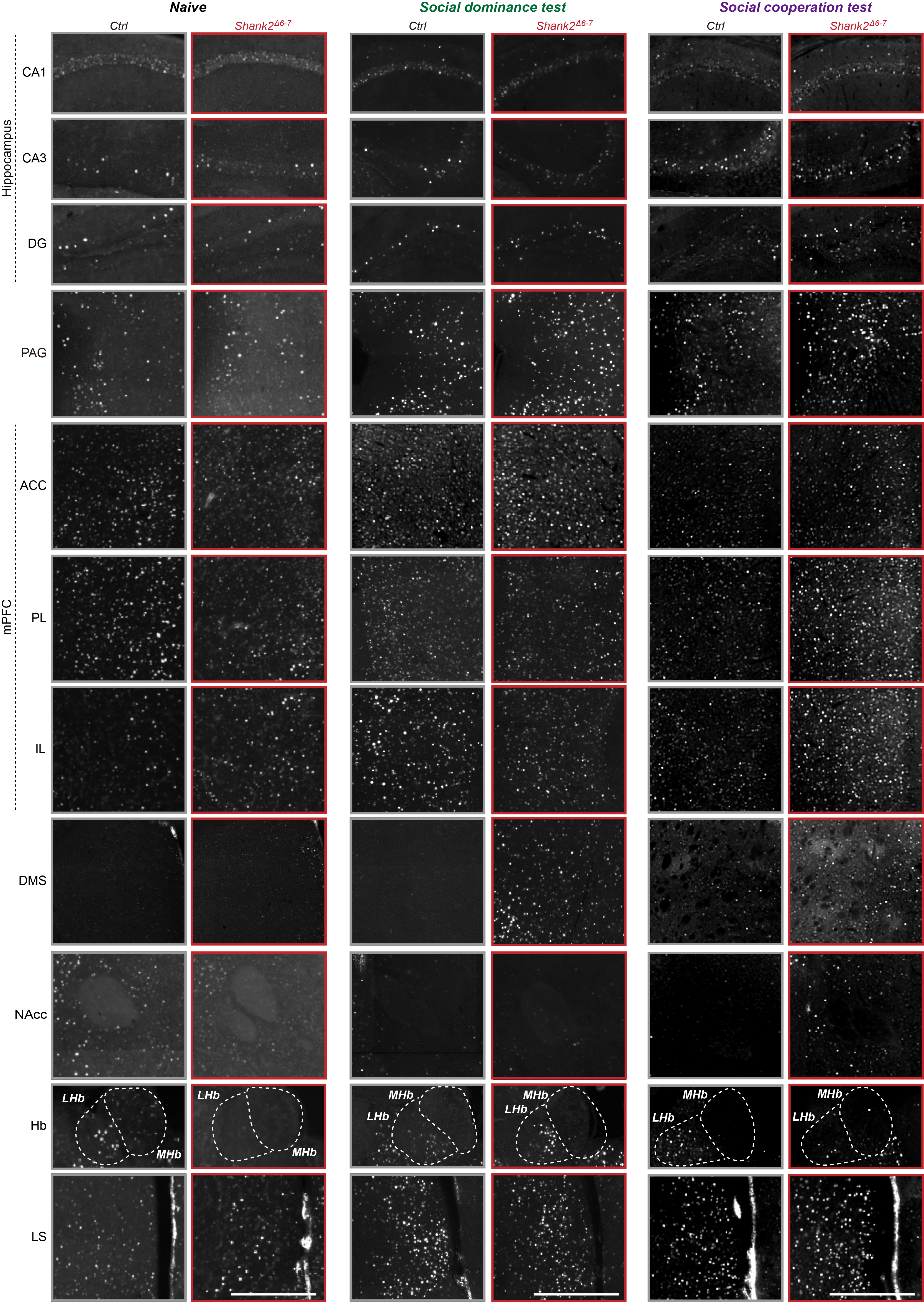


**Figure S3.** Representative images showing c-Fos immunostaining in the indicated brain regions of *Shank2^∆6-7^* mice. Quantified results are presented in **Figure 2b, 2d** and **2f**. (See also **Fig. S5** for quantification of c-Fos puncta intensity.) Scale bar: 20 μm.


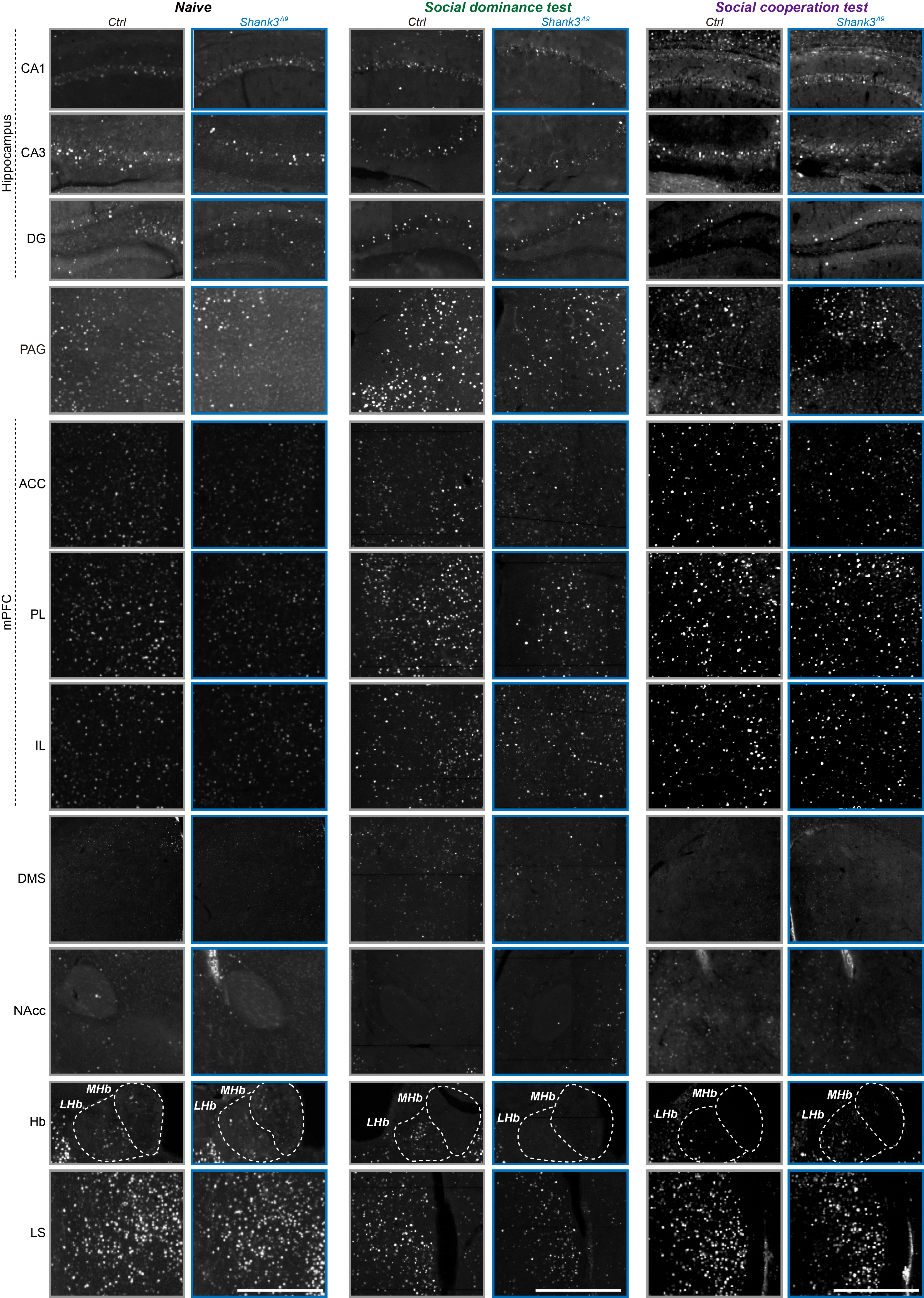


**Figure S4**. Representative images of c-Fos immunostaining in the indicated brain regions of *Shank3^∆9^* mice. Quantified results are presented in **Figure 2c, 2e** and **2g**. (See also **Fig. S5** for quantification of c-Fos puncta intensity.) Scale bar: 20 μm.


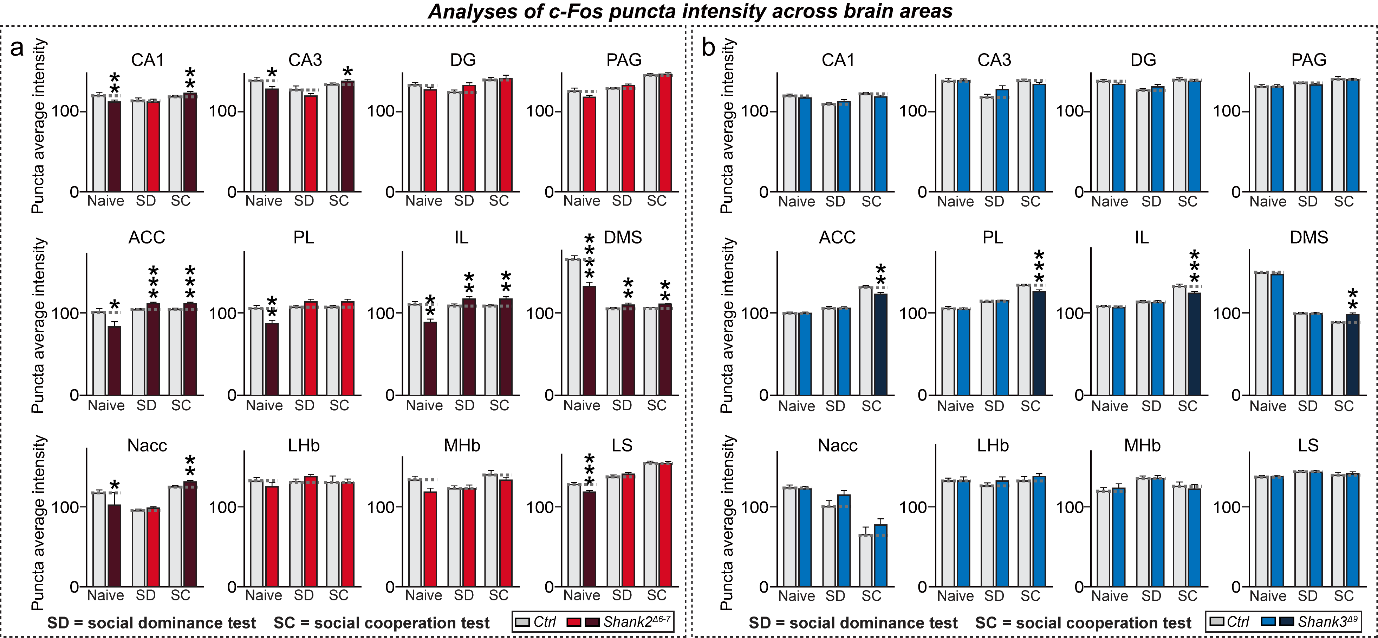


**Figure S5**. Quantitative analyses of c-Fos–positive puncta intensity across 12 brain regions of *Shank2^∆6 7^* and *Shank3^∆9^* mice. Quantification of activated neuron intensities of *Shank2^∆6-7^* and *Shank3^∆9^ mice* under naïve conditions or after the indicated behavioral experiences. Measurement of c-Fos puncta intensities presented in **Figure S3** (**a**) or **Figure S4** (**b**). Data are expressed as means ± SEMs (n is the number of mice analyzed; Ctrl/naive, n = 7–11 slices (3 mice), *Shank2^∆6-7^*/naïve, n = 7–11 slices (3 mice); Ctrl/SD, n = 10–12 slices (4 mice), *Shank2^∆6-7^*/SD, n = 11–12 slices (4 mice); Control/SC, n = 11–12 slices (4 mice), *Shank2^∆6-7^*/SC, n = 9–11 slices (4 mice); Control/naïve, n = 7–11 slices (3 mice), *Shank3^∆9^*/naïve, n = 9–16 slices (4 mice); Control/SD, n = 12 slices (4 mice), *Shank3^∆9^*/SD, n =11–12 slices (4 mice); Control/SC, n = 12 slices (4 mice); and *Shank3^∆9^*/SC, n = 12 slices (4 mice); ^*^*p* < 0.05, ^**^*p* < 0.01, ^***^*p* < 0.001, ^****^*p* < 0.0001; Mann-Whitney *U* test). Ctrl vs. *Shank2^∆6-7^*/naïve/CA1, *p* = 0.0021; Ctrl vs. *Shank2^∆6-7^*/naïve/CA3, *p* = 0.0242; Ctrl vs. *Shank2^∆6-7^*/naïve/DG, *p* = 0.1321; Ctrl vs. *Shank2^∆6-7^*/naïve/PAG, *p* = 0.1593; Ctrl vs. *Shank2^∆6-7^*/naïve/ACC, *p* = 0.0104; Ctrl vs. *Shank2^∆6-7^*/naïve/PL, *p* = 0.0041; Ctrl vs. *Shank2^∆6-7^*/naïve/IL, *p* = 0.0014; Ctrl vs. *Shank2^∆6-7^*/naïve/DMS, *p* < 0.0001; Ctrl vs *Shank2^∆6-7^*/naïve/Nacc, *p* = 0.1260; Ctrl vs *Shank2^∆6-7^*/naïve/LHb, *p* = 0.2370; Ctrl vs. *Shank2^∆6-7^*/naïve/MHb, *p* = 0.1636; Ctrl vs. *Shank2^∆6-7^*/naïve/LS, *p* = 0.0007; Ctrl vs. *Shank2^∆6-7^*/SD/CA1, *p* = 0.6297; Ctrl vs. *Shank2^∆6-7^*/SD/CA3, *p* = 0.0684; Ctrl vs. *Shank2^∆6-7^*/SD/DG, *p* = 0.0780; Ctrl vs. *Shank2^∆6-7^*/SD/PAG, *p* = 0.1431; Ctrl vs. *Shank2^∆6-7^*/SD/ACC, *p* = 0.0001; Ctrl vs. *Shank2^∆6-7^*/SD/PL, *p* = 0.1014; Ctrl vs. *Shank2^∆6-7^*/SD/IL, *p* = 0.0066; Ctrl vs. *Shank2^∆6-7^*/SD/DMS, *p* = 0.0048; Ctrl vs. *Shank2^∆6-7^*/SD/Nacc, *p* = 0.4071; Ctrl vs. *Shank2^∆6-7^*/SD/LHb, *p*=0.0651; Ctrl vs. *Shank2^∆6-7^*/SD/MHb, *p* = 0.8328; Ctrl vs. *Shank2^∆6-7^*/SD/LS, *p* = 0.2593; Ctrl vs. *Shank2^∆6-7^*/SC/CA1, *p* = 0.0043; Ctrl vs. *Shank2^∆6-7^*/SC/CA3, *p* = 0.0409; Ctrl vs. *Shank2^∆6-7^*/SC/DG , *p* = 0.6016; Ctrl vs. *Shank2^∆6-7^*/SC/PAG, *p* = 0.6016; Ctrl vs. *Shank2^∆6-7^*/SC/ACC, *p* = 0.0001; Ctrl vs. *Shank2^∆6-7^*/SC/PL, *p* = 0.1014; Ctrl vs. *Shank2^∆6-7^*/SC/IL, *p* = 0.0066; Ctrl vs. *Shank2^∆6-7^*/SC/DMS, *p* = 0.0048; Ctrl vs. *Shank3^∆9^*/SC/Nacc, *p* = 0.0018; Ctrl vs. *Shank2^∆6-7^*/SC/LHb, *p* = 0.7972; Ctrl vs. *Shank2^∆6-7^*/SC/MHb, *p* = 0.5190; Ctrl vs. *Shank2^∆6-7^*/SC/LS, *p* = 0.9323; Ctrl vs. *Shank3^∆9^*/naïve/CA1, *p* = 0.4114; Ctrl vs. *Shank3^∆9^*/naïve/CA3, *p* = 0.6273; Ctrl vs. *Shank3^∆9^*/naïve/DG, *p* = 0.2022; Ctrl vs. *Shank3^∆9^*/naïve/PAG, *p* = 0.6368; Ctrl vs. *Shank3^∆9^*/naïve/ACC, *p* = 0.7859; Ctrl vs. *Shank3^∆9^*/naïve/PL, *p* = 0.1896; Ctrl vs. *Shank3^∆9^*/naïve/IL, *p* = 0.4865; Ctrl vs. *Shank3^∆9^*/naïve/DMS, *p* = 0.5587; Ctrl vs. *Shank3^∆9^*/naïve/Nacc, *p* = 0.6065; Ctrl vs. *Shank3^∆9^*/naïve/LHb, *p* = 0.6556; Ctrl vs. *Shank3^∆9^*/naïve/MHb, *p* = 0.5414; Ctrl vs. *Shank3^∆9^*/naïve/LS, *p* = 0.4491; Ctrl vs. *Shank3^∆9^*/SD/CA1, *p* = 0.1978; Ctrl vs. *Shank3^∆9^*/SD/CA3, *p* = 0.1135; Ctrl vs. *Shank3^∆9^*/SD/DG, *p* = 0.0597; Ctrl vs. *Shank3^∆9^*/SD/PAG, *p* = 0.1932; Ctrl vs. *Shank3^∆9^*/SD/ACC, *p* = 0.6707; Ctrl vs. *Shank3^∆9^*/SD/PL, *p* = 0.1978; Ctrl vs. *Shank3^∆9^*/SD/IL, *p* = 0.5899; Ctrl vs. *Shank3^∆9^*/SD/DMS, *p* = 0.8461; Ctrl vs. *Shank3^∆9^*/SD/Nacc, *p* = 0.2014; Ctrl vs. *Shank3^∆9^*/SD/LHb, *p* = 0.1207; Ctrl vs. *Shank3^∆9^*/SD/MHb, *p* = 0.4776; Ctrl vs. *Shank3^∆9^*/SD/LS, *p* = 0.7553; Ctrl vs. *Shank3^∆9^*/SC/CA1, *p* = 0.0519; Ctrl vs. *Shank3^∆9^*/SC/CA3, *p* = 0.1277; Ctrl vs. *Shank3^∆9^*/SC/DG, *p* = 0.2415; Ctrl vs. *Shank3^∆9^*/SC/PAG, *p* = 0.3474; Ctrl vs. *Shank3^∆9^*/SC/ACC, *p* = 0.0011; Ctrl vs. *Shank3^∆9^*/SC/PL, *p* = 0.0003; Ctrl vs. *Shank3^∆9^*/SC/IL, *p* = 0.0002; Ctrl vs. *Shank3^∆9^*/SC/DMS, *p* = 0.0055; Ctrl vs. *Shank3^∆9^*/SC/Nacc, *p* = 0.1359; Ctrl vs. *Shank3^∆9^*/SC/LHb, *p* = 0.3474; Ctrl vs. *Shank3^∆9^*/SC/MHb, *p* = 0.4262; and Ctrl vs. *Shank3^∆9^*/SC/LS, *p* = 0.7125. See also **Table S3** for summary.

**Table S1. Details on statistics for behavioral analyses (Related to Figures 1 & S1).**

| **Mouse genotype** | **Behavior assay**  **(Figure)** | **Result** | **Number of mice** | **Statistics & p-values** |
| --- | --- | --- | --- | --- |
| *Shank2^∆6-7^* | Three-chamber test (**Figure S1b & S1c**) | Reduced social interaction | Ctrl = 11,  *Shank2^∆6-7^* = 11 | Mann-Whitney *U* test; social interaction (time spent in social activity test): Ctrl, *p* < 0.001; *Shank2^∆6-7^, p* = 0.2642; social novelty (time spent in social novelty test): Ctrl, *p* = 0.003; *Shank2^∆6-7^*, *p* =0.0002; social interaction (sniffing time in social activity test): Ctrl, *p =* 0.0008; *Shank2^∆6-7^, p* = 0.0723; social novelty (sniffing time in social novelty test): Ctrl, *p* = 0.0032; *Shank2^∆6-7^, p* = 0.0022 |
|  | Social dominance test (**Figure 1b**) | Dominant | Ctrl = 10,  *Shank2^∆6-7^* = 9 | Mann-Whitney *U* test; (left) D1: *p* = 0.0002, D2: *p* = 0.0034, D3: *p* = 0.0002 (left); (right) *p* = 0.0004 |
|  | Social cooperation test (**Figure 1d**) | Increased mutual rewards (statistically non-significant) | Ctrl, n = 6 (pairs);  *Shank2^∆6-7^*, n = 7 (pairs) | Two-way repeated measures ANOVA: genotype: F_(1,9)_ = 1.583, *p* = 0.2400; day: F_(3.763, 33.87)_ = 8.463, *p* < 0.0001; genotype × day: F_(11, 99)_ = 1.171, *p* = 0.3168) |
|  |  | Increased efficacy (statistically non-significant) |  | Two-way repeated measures ANOVA: genotype: F_(1,9)_ = 0.4601, *p* = 0.5146; day: F_(3.994,35.94)_ = 7.386, *p* = 0.0002; genotype × day: F_(11,99)_ = 0.6153, *p* = 0.8119) |
|  |  | No change in latency |  | Two-way repeated measures ANOVA: genotype: F_(1,9)_ = 0.04543, *p* = 0.8360; day: F_(4.166,37.49)_ = 5.841, *p* = 0.0008; genotype × day: F_(11,99)_ = 0.7166, p = 0.7201 |
|  |  | Increased activity |  | Two-way repeated measures ANOVA: genotype: F_(1,9)_=6.947, *p* = 0.0271; day: F_(3.226,29.04)_ = 7.820, *p* = 0.0004; genotype × day: F_(11,99)_ = 1.418, *p* = 0.1766) |
| *Shank3^∆9^* | Three-chamber test (**Figure S1e & S1f**) | Normal social interaction | Ctrl = 19,  *Shank3^∆9^* = 24 | Mann-Whitney *U* test;  social interaction (time spent in social activity test): Ctrl, *p=*0.0002;  *Shank3^∆9^, p=0.0005*;  social novelty (time spent in social novelty test): Ctrl, *p*<0.0001;  *Shank3^∆9^, p*<0.0001;  social interaction (sniffing time in social activity test): Ctrl, *p*<0.0001;  *Shank3^∆9^, p*<0.0001;  social novelty (sniffing time in social novelty test): Ctrl, *p*<0.0001;  *Shank3^∆9^, p=*0.0043 |
|  | Social dominance test (**Figure 1c**) | Subordinate | Ctrl = 11,  *Shank3^∆9^* = 13 | Mann-Whitney *U* test; (left) D1: *p* = 0.5785, D2: *p* = 0.0242, D3: *p* = 0.0186; (right) *p* = 0.0455 |
|  | Social cooperation test (**Figure 1e**) | Increased mutual rewards (statistically non-significant) | Ctrl, n = 6 (pairs);  *Shank3^∆9^*, n = 10 (pairs) | Two-way repeated measures ANOVA: genotype: F_(1,14)_ = 25.52, *p* = 0.0002; day: F_(4.437, 62.12)_ = 7.205, *p* < 0.0001; genotype × day: F_(11, 154)_ = 2.641, *p* = 0.0040) |
|  |  | Increased efficacy (statistically non-significant) |  | Two-way repeated measures ANOVA: genotype: F_(1,14)_ = 23.11, *p* = 0.0003; day: F_(5.091,71.27)_ = 5.113, *p* = 0.0004; genotype × day: F_(11,154)_ = 1.969, *p* = 0.0350) |
|  |  | No change in latency |  | Two-way repeated measures ANOVA: genotype: F_(1,14)_ = 16.28, *p* = 0.0012; day: F_(6.167,86.33)_ = 2.484, *p* = 0.0278; genotype × day: F_(11,154)_ = 0.7885, *p* = 0.6512) |
|  |  | Increased activity |  | Two-way repeated measures ANOVA: genotype: F_(11,209)_ = 1.305, *p* = 0.0031; day: F_(5.485,104.2)_ = 2.674, *p* = 0.0220; and genotype × day: F_(11,209)_ = 1.305, *p* = 0.2230) |

**Table S2. Details on statistics for c-Fos puncta density analyses (Related to Figure 2).**

| **Mouse genotype** | **Behavioral experience**  **(Figure)** | **Brain region** | **c-Fos density** | **Number of brain sections** | **Statistics & p-values** |
| --- | --- | --- | --- | --- | --- |
| *Shank2^∆6-7^* | Naïve  (**Figure 2b**) | CA1 | N.S. | Ctrl = 11, *Shank2^∆6-7^* = 10 | Mann-Whitney *U* test; *p* = 0.0845 |
|  |  | CA3 | N.S. | Ctrl = 11, *Shank2^∆6-7^* = 10 | Mann-Whitney *U* test; *p* = 0.6047 |
|  |  | DG | N.S. | Ctrl = 11, *Shank2^∆6-7^* = 10 | Mann-Whitney *U* test;  *p* = 0.2230 |
|  |  | PAG | N.S. | Ctrl = 12, *Shank2^∆6-7^* = 10 | Mann-Whitney *U* test; *p* = 0.9229 |
|  |  | ACC | Decreased | Ctrl = 11, *Shank2^∆6-7^* = 11 | Mann-Whitney *U* test; *p* = 0.0222 |
|  |  | PL | N.S. | Ctrl = 11, *Shank2^∆6-7^* = 11 | Mann-Whitney *U* test; *p* = 0.1987 |
|  |  | IL | Increased | Ctrl = 11, *Shank2^∆6-7^* = 11 | Mann-Whitney *U* test; *p* = 0.0008 |
|  |  | DMS | Increased | Ctrl = 24, *Shank2^∆6-7^* = 24 | Mann-Whitney *U* test; *p* = 0.0355 |
|  |  | Nacc | Decreased | Ctrl = 24, *Shank2^∆6-7^* = 24 | Mann-Whitney *U* test; *p* = 0.0218 |
|  |  | LHb | Decreased | Ctrl = 14, *Shank2^∆6-7^* = 11 | Mann-Whitney *U* test; *p* = 0.0020 |
|  |  | MHb | Decreased | Ctrl = 14, *Shank2^∆6-7^* = 11 | Mann-Whitney *U* test; *p* = 0.0053 |
|  |  | LS | N.S. | Ctrl = 12, *Shank2^∆6-7^* = 12 | Mann-Whitney *U* test; *p* = 0.0914 |
|  | After social dominance test  (**Figure 2d**) | CA1 | N.S. | Ctrl = 12, *Shank2^∆6-7^* = 12 | Mann-Whitney *U* test; *p* = 0.2189 |
|  |  | CA3 | N.S. | Ctrl = 12, *Shank2^∆6-7^* = 12 | Mann-Whitney *U* test; *p* = 0.6815 |
|  |  | DG | Increased | Ctrl = 12, *Shank2^∆6-7^* = 12 | Mann-Whitney *U* test; *p* = 0.0403 |
|  |  | PAG | Increased | Ctrl = 10, *Shank2^∆6-7^* = 10 | Mann-Whitney *U* test; *p* = 0.0295 |
|  |  | ACC | N.S. | Ctrl = 11, *Shank2^∆6-7^* = 11 | Mann-Whitney *U* test; *p* = 0.6505 |
|  |  | PL | N.S. | Ctrl = 11, *Shank2^∆6-7^* = 11 | Mann-Whitney *U* test; *p* = 0.7399 |
|  |  | IL | Decreased | Ctrl = 11, *Shank2^∆6-7^* = 11 | Mann-Whitney *U* test; *p* = 0.0129 |
|  |  | DMS | Increased | Ctrl = 24, *Shank2^∆6-7^* = 24 | Mann-Whitney *U* test; *p* = 0.0005 |
|  |  | Nacc | N.S. | Ctrl = 24, *Shank2^∆6-7^* = 24 | Mann-Whitney *U* test; *p* = 0.4492 |
|  |  | LHb | Increased | Ctrl = 19, *Shank2^∆6-7^* = 20 | Mann-Whitney *U* test; *p*=0.0464 |
|  |  | MHb | N.S. | Ctrl = 19, *Shank2^∆6-7^* = 20 | Mann-Whitney *U* test; *p* = 0.4567 |
|  |  | LS | N.S. | Ctrl = 12, *Shank2^∆6-7^* = 12 | Mann-Whitney *U* test; *p* = 0.2657 |
|  | After social cooperation test  (**Figure 2f**) | CA1 | Increased | Ctrl = 12, *Shank2^∆6-7^* = 9 | Mann-Whitney *U* test; *p* = 0.0278 |
|  |  | CA3 | N.S. | Ctrl = 12, *Shank2^∆6-7^* = 9 | Mann-Whitney *U* test; *p* = 0.0992 |
|  |  | DG | Increased | Ctrl = 12, *Shank2^∆6-7^* = 9 | Mann-Whitney *U* test; *p* = 0.0143 |
|  |  | PAG | Increased | Ctrl = 12, *Shank2^∆6-7^* = 9 | Mann-Whitney *U* test; *p* = 0.0013 |
|  |  | ACC | N.S. | Ctrl = 11, *Shank2^∆6-7^* = 11 | Mann-Whitney *U* test; *p* = 0.0977 |
|  |  | PL | Increased | Ctrl = 11, *Shank2^∆6-7^* = 11 | Mann-Whitney *U* test; *p* = 0.0134 |
|  |  | IL | Increased | Ctrl = 11, *Shank2^∆6-7^* = 11 | Mann-Whitney *U* test; *p* = 0.0019 |
|  |  | DMS | increased | Ctrl = 24, *Shank2^∆6-7^* = 24 | Mann-Whitney *U* test; *p* = 0.0058 |
|  |  | Nacc | increased | Ctrl = 12, *Shank2^∆6-7^* = 12 | Mann-Whitney *U* test; *p* = 0.0003 |
|  |  | LHb | Decreased | Ctrl = 11, *Shank2^∆6-7^* = 11 | Mann-Whitney *U* test; *p* = 0.0098 |
|  |  | MHb | Increased | Ctrl = 11, *Shank2^∆6-7^* = 11 | Mann-Whitney *U* test; *p* = 0.0482 |
|  |  | LS | N.S. | Ctrl = 12, *Shank2^∆6-7^* = 12 | Mann-Whitney *U* test; *p* = 0.4863 |
| *Shank3^∆9^* | Naïve  (**Figure 2c**) | CA1 | Increased | Ctrl = 9, *Shank3^∆9^* = 16 | Mann-Whitney *U* test; *p* = 0.0093 |
|  |  | CA3 | Decreased | Ctrl = 9, *Shank3^∆9^* = 16 | Mann-Whitney *U* test; *p* = 0.0225 |
|  |  | DG | N.S. | Ctrl = 9, *Shank3^∆9^* = 16 | Mann-Whitney *U* test; *p* = 0.5499 |
|  |  | PAG | N.S. | Ctrl = 9, *Shank3^∆9^* = 16 | Mann-Whitney *U* test; *p* = 0.6771 |
|  |  | ACC | N.S. | Ctrl = 11, *Shank3^∆9^* = 12 | Mann-Whitney *U* test; *p* = 0.0877 |
|  |  | PL | Decreased | Ctrl = 11, *Shank3^∆9^* = 12 | Mann-Whitney *U* test; *p* = 0.0214 |
|  |  | IL | N.S. | Ctrl = 11, *Shank3^∆9^* = 12 | Mann-Whitney *U* test; *p* = 0.0279 |
|  |  | DMS | Decreased | Ctrl = 13, *Shank3^∆9^* = 11 | Mann-Whitney *U* test; *p* = 0.0225 |
|  |  | Nacc | N.S. | Ctrl = 7, *Shank3^∆9^* = 9 | Mann-Whitney *U* test; *p* = 0.2523 |
|  |  | LHb | N.S. | Ctrl = 9, *Shank3^∆9^* = 11 | Mann-Whitney *U* test; *p* = 0.7103 |
|  |  | MHb | N.S. | Ctrl = 9, *Shank3^∆9^* = 11 | Mann-Whitney *U* test; *p* = 0.3693 |
|  |  | LS | N.S. | Ctrl = 9, *Shank3^∆9^* = 11 | Mann-Whitney *U* test; *p* = 0.9279 |
|  | After social dominance test  (**Figure 2e**) | CA1 | N.S. | Ctrl = 12, *Shank3^∆9^* = 12 | Mann-Whitney *U* test; *p* = 0.1978 |
|  |  | CA3 | N.S. | Ctrl = 12, *Shank3^∆9^* = 12 | Mann-Whitney *U* test; *p* = 0.1600 |
|  |  | DG | N.S. | Ctrl = 12, *Shank3^∆9^* = 12 | Mann-Whitney *U* test; *p* = 0.2189 |
|  |  | PAG | Decreased | Ctrl = 11, *Shank3^∆9^* = 11 | Mann-Whitney *U* test; *p* = 0.0006 |
|  |  | ACC | Decreased | Ctrl = 12, *Shank3^∆9^* = 12 | Mann-Whitney *U* test; *p* = 0.0018 |
|  |  | PL | Decreased | Ctrl = 12, *Shank3^∆9^* = 12 | Mann-Whitney *U* test; *p* = 0.0001 |
|  |  | IL | Decreased | Ctrl = 12, *Shank3^∆9^* = 12 | Mann-Whitney *U* test; *p* = 0.0014 |
|  |  | DMS | Decreased | Ctrl = 24, *Shank3^∆9^* = 24 | Mann-Whitney *U* test; *p* < 0.0001 |
|  |  | Nacc | N.S. | Ctrl = 9, *Shank3^∆9^* = 11 | Mann-Whitney *U* test; *p* = 0.4746 |
|  |  | LHb | Decreased | Ctrl = 12, *Shank3^∆9^* = 12 | Mann-Whitney *U* test; *p* = 0.0036 |
|  |  | MHb | N.S. | Ctrl = 12, *Shank3^∆9^* = 12 | Mann-Whitney *U* test; *p* = 0.0100 |
|  |  | LS | Decreased | Ctrl = 12, *Shank3^∆9^* = 12 | Mann-Whitney *U* test; *p* = 0.0001 |
|  | After social cooperation test  (**Figure 2g**) | CA1 | N.S. | Ctrl = 12, *Shank3^∆9^* = 12 | Mann-Whitney *U* test; *p* = 0.8428 |
|  |  | CA3 | N.S. | Ctrl = 12, *Shank3^∆9^* = 12 | Mann-Whitney *U* test; *p* = 0.1600 |
|  |  | DG | N.S. | Ctrl = 12, *Shank3^∆9^* = 12 | Mann-Whitney *U* test; *p* = 0.7125 |
|  |  | PAG | N.S. | Ctrl = 12, *Shank3^∆9^* = 12 | Mann-Whitney *U* test; *p* = 0.1600 |
|  |  | ACC | N.S. | Ctrl = 12, *Shank3^∆9^* = 12 | Mann-Whitney *U* test; *p* = 0.1432 |
|  |  | PL | N.S. | Ctrl = 12, *Shank3^∆9^* = 12 | Mann-Whitney *U* test; *p* = 0.1135 |
|  |  | IL | N.S. | Ctrl = 12, *Shank3^∆9^* = 12 | Mann-Whitney *U* test; *p* = 0.5137 |
|  |  | DMS | Increased | Ctrl = 24, *Shank3^∆9^* = 24 | Mann-Whitney *U* test; *p* = 0.0004 |
|  |  | Nacc | N.S. | Ctrl = 12, *Shank3^∆9^* = 12 | Mann-Whitney *U* test; *p* = 0.0841 |
|  |  | LHb | N.S. | Ctrl = 12, *Shank3^∆9^* = 12 | Mann-Whitney *U* test; *p* = 0.5137 |
|  |  | MHb | N.S. | Ctrl = 12, *Shank3^∆9^* = 12 | Mann-Whitney *U* test; *p* = 0.7985 |
|  |  | LS | N.S. | Ctrl = 12, *Shank3^∆9^* = 12 | Mann-Whitney *U* test; *p* = 0.6707 |

**Abbreviations:** N.S., not significant

**Table S3. Details on statistics for c-Fos puncta intensity analyses (Related to Figure S5).**

| **Mouse genotype** | **Behavioral experience**  **(Figure)** | **Brain region** | **c-Fos intensity** | **Number of brain sections** | **Statistics & p-values** |
| --- | --- | --- | --- | --- | --- |
| *Shank2^∆6-7^* | Naïve  (**Figure S5a**) | CA1 | Decreased | Ctrl = 11, *Shank2^∆6-7^* = 10 | Mann-Whitney *U* test; *p* = 0.0021 |
|  |  | CA3 | Decreased | Ctrl = 11, *Shank2^∆6-7^* = 10 | Mann-Whitney *U* test; *p* = 0.0242 |
|  |  | DG | N.S. | Ctrl = 11, *Shank2^∆6-7^* = 10 | Mann-Whitney *U* test;*p* = 0.1321 |
|  |  | PAG | N.S. | Ctrl = 12, *Shank2^∆6-7^* = 10 | Mann-Whitney *U* test; *p* = 0.1593 |
|  |  | ACC | Decreased | Ctrl = 11, *Shank2^∆6-7^* = 11 | Mann-Whitney *U* test; *p* = 0.0104 |
|  |  | PL | Decreased | Ctrl = 11, *Shank2^∆6-7^* = 11 | Mann-Whitney *U* test; *p* = 0.0041 |
|  |  | IL | Decreased | Ctrl = 11, *Shank2^∆6-7^* = 11 | Mann-Whitney *U* test; *p* = 0.0014 |
|  |  | DMS | Decreased | Ctrl = 24, *Shank2^∆6-7^* = 24 | Mann-Whitney *U* test; *p <0.0001* |
|  |  | Nacc | Decreased | Ctrl = 24, *Shank2^∆6-7^* = 24 | Mann-Whitney *U* test; *p* = 0.126 |
|  |  | LHb | N.S. | Ctrl = 14, *Shank2^∆6-7^* = 11 | Mann-Whitney *U* test; *p* = 0.2370 |
|  |  | MHb | N.S. | Ctrl = 14, *Shank2^∆6-7^* = 11 | Mann-Whitney *U* test; *p* = 0.1636 |
|  |  | LS | Decreased | Ctrl = 12, *Shank2^∆6-7^* = 12 | Mann-Whitney *U* test; *p* = 0.0007 |
|  | After social dominance test  (**Figure S5a**) | CA1 | N.S. | Ctrl = 12, *Shank2^∆6-7^* = 12 | Mann-Whitney *U* test; *p* = 0.6297 |
|  |  | CA3 | N.S. | Ctrl = 12, *Shank2^∆6-7^* = 12 | Mann-Whitney *U* test; *p* = 0.0684 |
|  |  | DG | N.S. | Ctrl = 12, *Shank2^∆6-7^* = 12 | Mann-Whitney *U* test; *p* = 0.0780 |
|  |  | PAG | N.S. | Ctrl = 10, *Shank2^∆6-7^* = 10 | Mann-Whitney *U* test; *p* = 0.01431 |
|  |  | ACC | Increased | Ctrl = 11, *Shank2^∆6-7^* = 11 | Mann-Whitney *U* test; *p* = 0.0001 |
|  |  | PL | N.S. | Ctrl = 11, *Shank2^∆6-7^* = 11 | Mann-Whitney *U* test; *p* = 0.1014 |
|  |  | IL | Increased | Ctrl = 11, *Shank2^∆6-7^* = 11 | Mann-Whitney *U* test; *p* = 0.0066 |
|  |  | DMS | Increased | Ctrl = 24, *Shank2^∆6-7^* = 24 | Mann-Whitney *U* test; *p* = 0.0048 |
|  |  | Nacc | N.S. | Ctrl = 24, *Shank2^∆6-7^* = 24 | Mann-Whitney *U* test; *p* = 0.4071 |
|  |  | LHb | N.S. | Ctrl = 19, *Shank2^∆6-7^* = 20 | Mann-Whitney *U* test; *p*=0.0651 |
|  |  | MHb | N.S. | Ctrl = 19, *Shank2^∆6-7^* = 20 | Mann-Whitney *U* test; *p* = 0.8328 |
|  |  | LS | N.S. | Ctrl = 12, *Shank2^∆6-7^* = 12 | Mann-Whitney *U* test; *p* = 0.2593 |
|  | After social cooperation (**Figure S5a**) | CA1 | Increased | Ctrl = 12, *Shank2^∆6-7^* = 9 | Mann-Whitney *U* test; *p* = 0.0043 |
|  |  | CA3 | Increased | Ctrl = 12, *Shank2^∆6-7^* = 9 | Mann-Whitney *U* test; *p* = 0.0409 |
|  |  | DG | N.S. | Ctrl = 12, *Shank2^∆6-7^* = 9 | Mann-Whitney *U* test; *p* = 0.6016 |
|  |  | PAG | N.S. | Ctrl = 12, *Shank2^∆6-7^* = 9 | Mann-Whitney *U* test; *p* = 0.6016 |
|  |  | ACC | Increased | Ctrl = 11, *Shank2^∆6-7^* = 11 | Mann-Whitney *U* test; *p* = 0.0001 |
|  |  | PL | N.S. | Ctrl = 11, *Shank2^∆6-7^* = 11 | Mann-Whitney *U* test; *p* = 0.1014 |
|  |  | IL | Increased | Ctrl = 11, *Shank2^∆6-7^* = 11 | Mann-Whitney *U* test; *p* = 0.0066 |
|  |  | DMS | Increased | Ctrl = 24, *Shank2^∆6-7^* = 24 | Mann-Whitney *U* test; *p* = 0.0048 |
|  |  | Nacc | Increased | Ctrl = 12, *Shank2^∆6-7^* = 12 | Mann-Whitney *U* test; *p* = 0.0018 |
|  |  | LHb | N.S. | Ctrl = 11, *Shank2^∆6-7^* = 11 | Mann-Whitney *U* test; *p* = 0.7972 |
|  |  | MHb | N.S. | Ctrl = 11, *Shank2^∆6-7^* = 11 | Mann-Whitney *U* test; *p* = 0.5190 |
|  |  | LS | N.S. | Ctrl = 12, *Shank2^∆6-7^* = 12 | Mann-Whitney *U* test; *p* = 0.9323 |
| *Shank3^∆9^* | Naïve  (**Figure S5b**) | CA1 | N.S. | Ctrl = 9, *Shank3^∆9^* = 16 | Mann-Whitney *U* test; *p* = 0.4114 |
|  |  | CA3 | N.S. | Ctrl = 9, *Shank3^∆9^* = 16 | Mann-Whitney *U* test; *p* = 0.6273 |
|  |  | DG | N.S. | Ctrl = 9, *Shank3^∆9^* = 16 | Mann-Whitney *U* test; *p* = 0.2022 |
|  |  | PAG | N.S. | Ctrl = 9, *Shank3^∆9^* = 16 | Mann-Whitney *U* test; *p* = 0.6368 |
|  |  | ACC | N.S. | Ctrl = 11, *Shank3^∆9^* = 12 | Mann-Whitney *U* test; *p* = 0.7859 |
|  |  | PL | N.S. | Ctrl = 11, *Shank3^∆9^* = 12 | Mann-Whitney *U* test; *p* = 0.1896 |
|  |  | IL | N.S. | Ctrl = 11, *Shank3^∆9^* = 12 | Mann-Whitney *U* test; *p* = 0.4865 |
|  |  | DMS | N.S. | Ctrl = 13, *Shank3^∆9^* = 11 | Mann-Whitney *U* test; *p* = 0.5587 |
|  |  | Nacc | N.S. | Ctrl = 7, *Shank3^∆9^* = 9 | Mann-Whitney *U* test; *p* = 0.6065 |
|  |  | LHb | N.S. | Ctrl = 9, *Shank3^∆9^* = 11 | Mann-Whitney *U* test; *p* = 0.6556 |
|  |  | MHb | N.S. | Ctrl = 9, *Shank3^∆9^* = 11 | Mann-Whitney *U* test; *p* = 0.5414 |
|  |  | LS | N.S. | Ctrl = 9, *Shank3^∆9^* = 11 | Mann-Whitney *U* test; *p* = 0.4491 |
|  | After social dominance test  (**Figure S5b**) | CA1 | N.S. | Ctrl = 12, *Shank3^∆9^* = 12 | Mann-Whitney *U* test; *p* = 0.1978 |
|  |  | CA3 | N.S. | Ctrl = 12, *Shank3^∆9^* = 12 | Mann-Whitney *U* test; *p* = 0.1135 |
|  |  | DG | N.S. | Ctrl = 12, *Shank3^∆9^* = 12 | Mann-Whitney *U* test; *p* = 0.0597 |
|  |  | PAG | N.S. | Ctrl = 11, *Shank3^∆9^* = 11 | Mann-Whitney *U* test; *p* = 0.1932 |
|  |  | ACC | N.S. | Ctrl = 12, *Shank3^∆9^* = 12 | Mann-Whitney *U* test; *p* = 0.6707 |
|  |  | PL | N.S. | Ctrl = 12, *Shank3^∆9^* = 12 | Mann-Whitney *U* test; *p* = 0.1978 |
|  |  | IL | N.S. | Ctrl = 12, *Shank3^∆9^* = 12 | Mann-Whitney *U* test; *p* = 0.5899 |
|  |  | DMS | N.S. | Ctrl = 24, *Shank3^∆9^* = 24 | Mann-Whitney *U* test; *p = 0.8461* |
|  |  | Nacc | N.S. | Ctrl = 9, *Shank3^∆9^* = 11 | Mann-Whitney *U* test; *p* = 0.2014 |
|  |  | LHb | N.S, | Ctrl = 12, *Shank3^∆9^* = 12 | Mann-Whitney *U* test; *p* = 0.1207 |
|  |  | MHb | N.S. | Ctrl = 12, *Shank3^∆9^* = 12 | Mann-Whitney *U* test; *p* = 0.4776 |
|  |  | LS | N.S. | Ctrl = 12, *Shank3^∆9^* = 12 | Mann-Whitney *U* test; *p* = 0.7553 |
|  | After social cooperation test  (**Figure S5b**) | CA1 | N.S. | Ctrl = 12, *Shank3^∆9^* = 12 | Mann-Whitney *U* test; *p* = 0.0519 |
|  |  | CA3 | N.S. | Ctrl = 12, *Shank3^∆9^* = 12 | Mann-Whitney *U* test; *p* = 0.1277 |
|  |  | DG | N.S. | Ctrl = 12, *Shank3^∆9^* = 12 | Mann-Whitney *U* test; *p* = 0.2415 |
|  |  | PAG | N.S. | Ctrl = 12, *Shank3^∆9^* = 12 | Mann-Whitney *U* test; *p* = 0.3474 |
|  |  | ACC | Decreased | Ctrl = 12, *Shank3^∆9^* = 12 | Mann-Whitney *U* test; *p* = 0.0011 |
|  |  | PL | Decreased | Ctrl = 12, *Shank3^∆9^* = 12 | Mann-Whitney *U* test; *p* = 0.0003 |
|  |  | IL | Decreased | Ctrl = 12, *Shank3^∆9^* = 12 | Mann-Whitney *U* test; *p* = 0.0002 |
|  |  | DMS | Increased | Ctrl = 24, *Shank3^∆9^* = 24 | Mann-Whitney *U* test; *p* = 0.0055 |
|  |  | Nacc | N.S. | Ctrl = 12, *Shank3^∆9^* = 12 | Mann-Whitney *U* test; *p* = 0.1359 |
|  |  | LHb | N.S. | Ctrl = 12, *Shank3^∆9^* = 12 | Mann-Whitney *U* test; *p* = 0.3474 |
|  |  | MHb | N.S. | Ctrl = 12, *Shank3^∆9^* = 12 | Mann-Whitney *U* test; *p* = 0.4262 |
|  |  | LS | N.S. | Ctrl = 12, *Shank3^∆9^* = 12 | Mann-Whitney *U* test; *p* = 0.7125 |

**Abbreviations:** N.S., not significant
